# Evofosfamide Enhances Sensitivity of Breast Cancer Cells to Apoptosis and Natural-Killer-Cell-Mediated Cytotoxicity Under Hypoxic Conditions

**DOI:** 10.3390/cancers17121988

**Published:** 2025-06-14

**Authors:** Shubhankar Das, Goutham Hassan Venkatesh, Walid Shaaban Moustafa Elsayed, Raefa Abou Khouzam, Ayda Shah Mahmood, Husam Hussein Nawafleh, Nagwa Ahmed Zeinelabdin, Rania Faouzi Zaarour, Salem Chouaib

**Affiliations:** 1 Thumbay Research Institute for Precision Medicine, Gulf Medical University, Ajman 4184, United Arab Emirates; dr.shubhankar@gmu.ac.ae (S.D.); gouthamhv@gmail.com (G.H.V.); dr.raefa@gmu.ac.ae (R.A.K.); ayda@gmu.ac.ae (A.S.M.); husam@gmu.ac.ae (H.H.N.); nagwa@gmu.ac.ae (N.A.Z.); dr.rania@gmu.ac.ae (R.F.Z.); 2 College of Dentistry, Gulf Medical University, Ajman 4184, United Arab Emirates; dr.walidshaaban@gmu.ac.ae; 3 INSERM UMR 1186, Integrative Tumor Immunology and Immunotherapy, Gustave Roussy, University Paris-Saclay, 94805 Villejuif, France

**Keywords:** TH302, hypoxic tumors, NK-92 cells, cancer sensitization

## Abstract

**Background/objectives:** Hypoxia in the tumor microenvironment is linked to aggressiveness, epithelial–mesenchymal transition, metastasis, and therapy resistance. Targeting hypoxia to enhance antitumor immunity is crucial for overcoming therapeutic resistance. Here, we investigated the ability of Evofosfamide, a prodrug that gets activated under hypoxic conditions, to sensitize breast cancer cells to cell death. Evofosfamide is converted into bromo-isophosphoramide mustard, a potent DNA cross-linking agent that is expected to enhance the killing of cancer cells under hypoxic conditions, where these cells typically exhibit resistance. **Methods:** Representative breast cancer cell lines, MCF-7 and MDA-MB-231, were treated with Evofosfamide under normoxia and hypoxia. Changes in cell viability and the mechanism of cell death were measured using neutral red dye uptake, Annexin-FITC/propidium iodide staining, and Western blot analysis of markers—PARP1 and caspase 3/7. We tested Evofosfamide’s ability to counteract hypoxic suppression of type I Interferon signaling genes using quantitative PCR (qPCR), as well as its capacity to trigger natural killer (NK)-cell-mediated cytotoxicity. **Results:** Evofosfamide enhanced cell killing in both MCF-7 and MDA-MB-231 cells under hypoxic conditions compared to normoxic conditions. Cell killing was accompanied by increased cellular reactive oxygen species (ROS), diminished mitochondrial membrane potential, and induction of apoptosis, as demonstrated by the fragmentation or laddering of genomic DNA, the activation of caspase 3/7, and the cleavage of PARP. qPCR analysis revealed that Evofosfamide was capable of restoring type I interferon signaling in hypoxic breast cancer cells, leading to the subsequent cytolytic activity of NK cells against the tumor cells. **Conclusions:** Thus, conditioning the breast cancer cells with Evofosfamide resulted in enhanced cell killing under hypoxia, further underscoring its potential as a sensitizer to target hypoxia-driven tumors.

## Simple Summary

Resistance to treatment in breast cancer may arise from various factors, notably, the hypoxic tumor microenvironment. In hypoxic conditions, cancer cells stabilize hypoxia-inducible factors, with HIF-1α playing a pivotal role in conferring metabolic plasticity and facilitating immune evasion in cancer cells. Accordingly, it is essential to develop treatment strategies that effectively target cancer cells in hypoxic environments. The current in vitro study assessed the efficacy of Evofosfamide, a hypoxia-activated prodrug, against breast cancer cell lines and elucidated its mechanism of action under hypoxic conditions. The findings illustrate that breast cancer cell lines treated with Evofosfamide can be sensitized to apoptotic cell death under such conditions. Furthermore, Evofosfamide-exposed cells in hypoxia were observed to exhibit increased sensitivity to natural-killer-cell-mediated cytotoxicity, attributable to the preservation of type I interferon signaling, which is otherwise diminished in hypoxic environments. This indicates the tumor-immunomodulatory potential of Evofosfamide.

## 1. Introduction

Breast cancer (BC) is a prevalent cancer that significantly contributes to cancer-related deaths worldwide. Despite substantial progress in understanding the onset and progression of breast cancer, current treatment outcomes remain suboptimal. Patient-related factors, including age, ethnicity, and tumor location, as well as cancer-related elements such as driver mutations, clonal evolution, and the tumor microenvironment (TME), are critical in determining treatment outcomes and are associated with resistance [1]. In BC, the activation and stabilization of hypoxia-inducible factor (HIF-1α) due to low oxygen tension within the tumor microenvironment (TME) play a crucial role in determining the severity and poor prognosis of the disease [2]. HIF-1α and its target genes enable metabolic flexibility, leading to epigenetic changes in BC that contribute to therapy resistance and facilitate dissemination to distant sites.

Small-molecule inhibitors for HIF1α have been tested in preclinical models. Still, outcomes have not been satisfactory in clinical settings, which warrants the development of better strategies to manage hypoxia-driven tumors [3]. In this context, several hypoxia-activated prodrugs (HAPs) have been tested against various cancers [4]. Under low oxygen tension, HAP converts into byproducts that interact with genomic and mitochondrial DNA, inducing cell death by causing DNA damage, replication stress, cell cycle arrest, and apoptosis [5]. Evofosfamide (Evofos) or TH302 is a HAP that releases a bromo-iso-phosphoramide mustard in the presence of cellular oxidoreductases under hypoxic conditions, acting as a potent alkylating genotoxin that leads to DNA damage and cell death [6]. Evofos has been evaluated as a treatment for various cancer types, both as a monotherapy and in combination with agents such as cisplatin, doxorubicin, temozolomide, and radiation therapy [7,8]. Evofos has demonstrated promising outcomes in in vitro and in vivo studies [8,9] as well as in Phase I/II trials [10,11]. Although Evofos failed to clear Phase III trials due to adverse effect including gastrointestinal syndrome, skin reactions, and abnormalities in blood cells and inadequate overall survival in patients [12,13], Jayaprakash and colleagues demonstrated in a preclinical prostate cancer model that Evofos targets hypoxia, restores T-cell infiltration, increases T-cell proliferation within the TME, and sensitizes tumors to immune checkpoint blockade [14]. It has been established that hypoxia modulates the immune response within the TME. Previous studies suggest that a hypoxic TME hinders the infiltration and activation of cytotoxic T and NK cells while promoting the expansion of immune-suppressive myeloid-derived suppressor cells and regulatory T cells [15]. Tumor hypoxia can also induce phenotypic and genetic heterogeneity, thereby affecting clonal evolution and anti-tumor immunity [15]. It has been reported that hypoxia reduces cellular levels of cyclic GMP–AMP synthase (cGAS), lowering the activation of the stimulator of interferon genes (STING) [16]. While cGAS-STING activation regulates type I interferon signaling, enhancing the innate immune response and anti-tumor immunity, downregulation of cGAS-STING promotes immune escape in cancer cells [16,17].

Thus, activating type I IFNs in hypoxic tumors could have significant implications for tumor immunology and the advancement of combinatorial therapies. In this study, we examined the ability of Evofos to induce cell death in breast cancer cells under hypoxic conditions. Since Evofos treatment is associated with DNA damage under hypoxia, we anticipated that this may further contribute to the cGAS-STING pathway and elicit type I IFN signaling cascades, enhancing the anti-tumor activity of NK cells and thereby improving treatment outcomes in breast cancer.

## 2. Materials and Methods

### 2.1. Chemicals, Reagents, and Antibodies

Evofosfamide (Cas No. 918633-87-1), 2′,7′-Dichlorofluorescin Diacetate (DCFDA; Cas No. 4091-99-0), and propidium iodide (Cas No. 25535-16-4) were obtained from Sigma-Aldrich, St. Louis, MO, USA. Tetramethylrhodamine, methyl ester (TMRM; ab275547) was acquired from Abcam Limited, Cambridge, UK. All other reagents, unless otherwise specified, were sourced from Life Technologies, Carlsbad, CA, USA. The primary and secondary antibodies used in this study are listed in Supplementary Table S1.

### 2.2. Cell Lines

The human-breast-tissue-derived cell lines MCF-7 (NCI-DTP Cat# MCF7, RRID: CVCL_0031), MDA-MB-231 (NCI-DTP Cat# MDA-MB-231, RRID: CVCL_0062), and the natural killer cell line NK-92 (a kind gift from Prof. Fathia Mami Chouaib, INSERM, Gustave Roussy, France) were utilized in this study. All cell lines were cultured in RPMI 1640 GlutaMax medium supplemented with 10% Fetal Bovine Serum (FBS), 1 mM Sodium pyruvate, and antibiotics, including 100 U/mL Penicillin and 100 µg/mL Streptomycin. To maintain NK-92, the growth medium was supplemented with 100 U/mL human recombinant interleukin-2 (IL-2). The cell lines were routinely sub-cultured and maintained at 60–70% confluence in a 5% CO_2_ incubator (ESCO Cell Culture incubator, Horsham, PA, USA). Cells were tested for Mycoplasma infection by PCR every two weeks.

### 2.3. Preparation of Evofosfamide (Evofos)

Evofos was dissolved at a concentration of 50 mM in anhydrous cell culture-grade dimethyl sulfoxide and stored at −20 °C in small aliquots of 5 µL. Each time, a new aliquot of Evofos was diluted in cell culture medium according to the experimental conditions.

### 2.4. Treatment with Evofos Under Normoxia and Hypoxia

Cells treated with or without Evofos were incubated in either normoxic or hypoxic conditions. Cultures maintained in a humidified 5% CO_2_ incubator were classified as normoxic. For hypoxic conditions, cells were grown in a humidified Whitley H35 Hypoxystation (Don Whitley Scientific Limited) at 37 °C with an atmospheric composition of 95% N_2_, 5% CO_2_, and 1% O_2_, which is referred to as hypoxia. Untreated cells under normoxia served as experimental control.

### 2.5. Neutral Red Assay for Cell Viability

This assay was performed as described by Repetto et al. (2008) [18]. Briefly, MDA-MB-231 and MCF-7 cells were seeded at a density of 7000 cells per well in a 96-well plate and allowed to attach overnight. The following day, cells were treated with various concentrations of Evofosfamide and incubated under normoxia or hypoxia (1% O_2_) for 72 h. After drug treatment, cells were stained with neutral red dye prepared in complete medium (at a concentration of 100 µg/mL) and further incubated for 4 h. Next, the dye-containing medium was discarded, and 100 µL of solubilizing agent was added to each well to dissolve the accumulated dye inside the cells. The absorbance was measured at 540 nm using a plate reader, and the cell viability was calculated using the following expression.

From the results obtained in this set of experiments, we selected 1.2 and 4 µM of Evofosfamide to treat MCF-7 and MDA-MB-231 cells for all the experiments mentioned below. Both cell lines were seeded at a density of 3 × 10^5^ cells in 4 mL of growth medium in 60 mm cell culture dishes.

### 2.6. Measurement of Cell Death by Annexin FITC/Propidium Iodide (PI) Staining

This assay was conducted following the manufacturer’s protocol (eBioscience™ Annexin V Apoptosis Detection Kit; #88-8005-74). Cells were treated with Evofosfamide and incubated under normoxia or 1% hypoxia for 72 h. Afterward, they were harvested via trypsinization, and the cell pellets were resuspended in 1× binding buffer provided in the kit. The cell suspension was treated with 5 µL of Annexin-FITC and 5 µL of propidium iodide and incubated in the dark for 10 min. Next, a minimum of 10,000 events were acquired using the Bio-Rad S3e flow cytometer, and the data obtained were analyzed using FCS Express 7 software. A bivariate analysis was conducted to represent the data, with the *x*-axis showing Annexin-FITC-positive cells and the *y*-axis demonstrating PI-positive cells.

### 2.7. Estimation of Changes in Cell Cycle Arrest Using PI Staining

Cells treated with Evofosfamide were maintained under normoxic or hypoxic conditions (1% O_2_) and harvested after 48 h post-treatment. Cells were collected by trypsinization, washed with ice-cold sterile PBS (pH 7.4) to remove media residues, and fixed in 70% ethanol prepared in sterile water. After fixation, the cells were rinsed in PBS (pH 7.4) to ensure complete removal of the fixative. Cell pellets were resuspended in the remaining PBS and treated with RNase A for 3 h, followed by incubation in a water bath at 37 °C. Subsequently, cells were stained with 10 µL of propidium iodide (1 mg/mL) and incubated on ice in the dark for 30 min. Finally, a minimum of 10,000 events were acquired using the Bio-Rad S3e flow cytometer, and the collected data were analyzed with FCS Express 7 software.

### 2.8. Measurement of Cellular ROS and Mitochondrial Membrane Potential

Cellular ROS was measured using the DCFDA method, as described earlier [19]. MCF7 and MDA-MB-231 cells were treated with Evofos and incubated under normoxic or hypoxic conditions for up to 48 h. Next, the cells were loaded with 5 µM of DCFDA in serum-free medium for 20 min and then harvested by mild trypsinization. The cells were collected in 0.1% BSA in PBS (pH 7.4) and analyzed by flow cytometry using 488 nm/525 nm as excitation/emission; the resulting data were analyzed using FCS Express 7 software. To measure changes in mitochondrial membrane potential, the cells were loaded with 100 nM TMRM prepared in serum-free medium and further incubated for 20 min. Next, the cells were collected by mild trypsinization, resuspended in 0.1% BSA in PBS (pH 7.4), and analyzed by flow cytometry using 544 nm/574 nm as excitation/emission; the resulting data were analyzed using FCS Express 7 software [20].

### 2.9. TCGA Analysis

Transcriptomic and clinical data from 1050 breast cancer patients in the BRCA TCGA PanCancer dataset were obtained from cbioportal (https://www.cbioportal.org/; accessed on 16 January 2021). The 51-gene Buffa hypoxia signature [21] and the 18-gene Expanded Immune Signature [22] were employed to categorize the data according to hypoxia score and immune score, both separately and in combination. The scoring system utilized to evaluate the tumors of patients was based on our previous research [23,24]. In summary, medians were initially calculated for each gene within a given signature, followed by the transformation of each patient’s expression values for these genes into −1 or 1, depending on whether the gene exhibited lower or higher expression relative to its median. Consequently, the tumor’s score was computed as the aggregate of these transformed values, and patients were classified as having a high hypoxia score (HS) or a high immune score (IS) if their scores exceeded zero; otherwise, they were classified as having a low IS or a low HS. Survival analysis was conducted using RStudio V1.3 (RStudio, PBC, USA), utilizing the survive package, and the area under the curve was determined via the survival ROC. Overall survival (OS) and disease-specific survival (DSS) were compared between the low- and high-scoring groups for up to 10 years through Kaplan–Meier survival analysis. The log-rank test was employed to evaluate the null hypothesis of equality of survival distributions, with a *p*-value of <0.05 deemed statistically significant.

### 2.10. Changes in the Expression of Type-I-IFN-Associated Gene Expression

Post-treatment with Evofosfamide, MCF7, and MDA-MB-231 cells were incubated in normoxia and hypoxia for 24 h. Following this, total RNA was isolated from the cells using the easy-BLUE Total RNA Extraction Kit (iNtRON Biotechnologies) according to the manufacturer’s protocol. RNA samples were quantified using a Nanodrop. cDNA was reverse-transcribed with 1 μg of total RNA using a High-Capacity cDNA Reverse Transcription Kit (Applied Biosystems, Thermo Fisher, Waltham, MA, USA). qPCR was performed using the Maxima SYBR Green/ROX qPCR Master Mix (Thermo Scientific, USA) on a Biorad Real-Time thermocycler (CFX384 Touch Real-Time PCR Detection System, Biorad, CA, USA), and relative mRNA expression was normalized to β-actin. PCR primers used in the study are described in Supplementary Table S2.

### 2.11. Celltracker Green/Propidium Iodide Staining for Determining NK-92-Cell-Mediated Cytotoxicity Using Flow Cytometry

Natural-killer-cell-mediated cytotoxicity assays can be performed in vitro using the NK-92 cell line, which serves as a surrogate for natural killer cells. This method involves staining the target cells with CellTracker dye, followed by incubation with NK-92 cells (effector cells) and co-staining with propidium iodide. The extent of cytotoxicity is determined by flow cytometry, following the protocol described by Kim and coworkers (2007) [25], with minor modifications. Initially, we established the protocol using K562 cells as they are suitable targets for NK-92 cells and provided a detailed description of the flow cytometric acquisition (Supplementary Figure S1).

For the experiment, MCF-7 and MDA-MB-231 cells were treated with Evofos and incubated in either normoxic or hypoxic conditions for 24 h. Afterward, they were assayed for NK-92-cell-mediated cytotoxicity. Briefly, after treatment with Evofos, cells were collected in sterile 15 mL tubes and labeled with 1 mL of 40 nM CellTracker Green in sterile PBS containing 0.5% FBS for 15 min at 37 °C. We found that 40 nM is the optimal dye concentration for our cell lines, minimizing channel bleeding from the cell tracker probe. After staining, cells were rinsed twice with 10 volumes of complete medium to remove any unbound CellTracker Green probe. They were then counted and cocultured for 4 h at 37 °C with NK-92 cells (ratio of 1:15; MCF-7 or MDA-MB-231: NK-92) in complete medium supplemented with 200 units of recombinant IL-2. Next, cells were loaded with 10 µL of 50 µg/mL propidium iodide and incubated on ice for 30 min to stain the dying or dead cells. Cells were acquired by flow cytometry. The percentage of cytotoxicity induced by NK-92 was determined by measuring the population of CellTracker Green-positive cells that were also positive for propidium iodide.

### 2.12. Preparation of Cell Lysate for Western Blot

Cells were treated with Evofosfamide and incubated under normoxic or hypoxic conditions. They were then harvested and lysed in ice-cold RIPA lysis buffer containing 1% SDS, supplemented with 1 mM PMSF, 1 mM sodium orthovanadate, and a 1× protease inhibitor cocktail. Next, the lysates were sonicated and centrifuged at 13,000 rpm for 15 min at 4 °C. The protein concentration was measured using the standard BCA method (Pierce BCA Protein Assay Kit, ThermoFisher Scientific, USA). Fifty micrograms of protein sample were mixed with Laemmli buffer, denatured at 95 °C for 5 min, and resolved in 8–12% SDS-polyacrylamide gels. The resolved proteins were transferred to a 0.2 µ nitrocellulose membrane (Amersham Protran, Sigma, USA), blocked with 5% skimmed milk prepared with 0.1% Tween-20 in Tris-buffered saline (TBST) for 1 h at room temperature. The blocked membranes were washed with TBST and probed with the respective primary antibody overnight at 4 °C with mild rocking. Next, a secondary antibody was added, and the membranes were incubated at room temperature for 1 h. Membranes were then developed using the SuperSignal West Pico PLUS Chemiluminescent Substrate kit (Thermo Fisher Scientific, USA) on an iBright CL750 Imaging System (Thermo Fisher Scientific, USA). Changes in the expression of cGAS/phsopho-STING^Ser366^, and HIF-1α were measured after 24 h of treatment, whereas apoptosis-related proteins and p21 levels were measured at 48 h post-treatment.

### 2.13. Statistical Analysis

Statistical analysis for all the assays was performed on GraphPad Prism, version 5.0 (GraphPad Software, San Diego, CA, USA). For all comparisons, either an unpaired (two-tailed) *t*-test or one-way analysis of variance (ANOVA) with Bonferroni’s post hoc test was performed. A *p*-value of <0.05 was considered statistically significant. The results are represented as mean ± SD for three independent experiments. For TCGA analysis, the log-rank test was employed to evaluate the null hypothesis of equality of survival distributions, with a *p*-value of <0.05 deemed statistically significant.

## 3. Results

### 3.1. The Evofos-Induced Breast Cancer Cell Death Under Hypoxia Is Associated with Alterations in Cell Cycle, DNA Damage, and Apoptotic Cell Death

**Figure 1:**
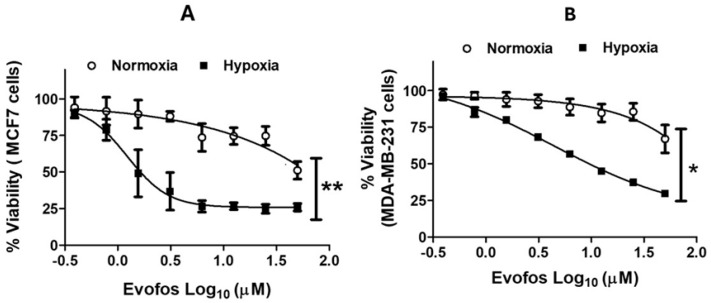
Treatment with Evofosfamide led to decreased cell viability under hypoxic conditions compared to normoxia in breast cancer cell lines, MCF-7 and MDA-MB-231. The graphs display non-linear regression curves for MCF-7 (**A**) and MDA-MB-231 (**B**), demonstrating viability after treatment with various concentrations of Evofos (0.39 µM–50 µM) in both normoxic and hypoxic (1% O_2_) environments, measured at 72 h. Data presented as mean ± SD; N = 3. * *p* < 0.05; ** *p* < 0.01 when compared between normoxia and hypoxia as determined by unpaired (two-tailed) *t*-test.

We assessed the viability of MCF7 and MDA-MB-231 cells treated with Evofosfamide under normoxia and hypoxia using a neutral red dye uptake assay. Cells exposed to varying concentrations of Evofosfamide (0.39–50 µM) for a duration of 72 h demonstrated increased cell death under hypoxic conditions compared to normoxic conditions. Under normoxia, both MCF7 and MDA-MB-231 showed a decline in cell viability, but only at higher concentrations of Evofos. Evofos treatment under 1% O_2_ hypoxia increased cell death. The IC50 values for MCF-7 and MDA-MB-231 under hypoxia were calculated to be 1.56 and 4.37 µM, respectively (Figure 1A,B).

**Figure 2:**
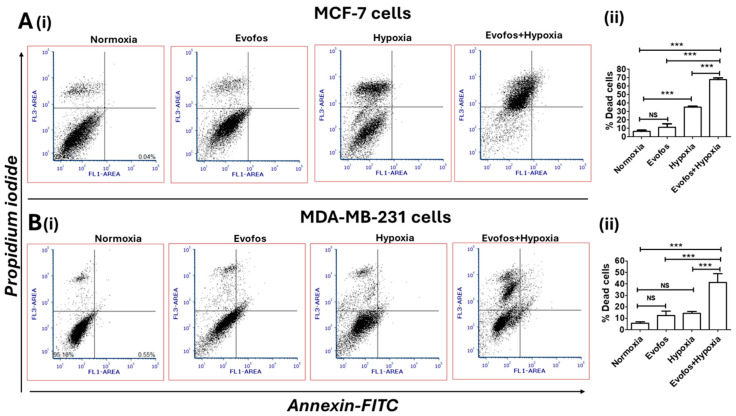
Evofos treatment increased cell death in both MCF-7 and MDA-MB-231 cells under hypoxia. (**A**) (**i**) shows bivariate analysis of Annexin-FITC/ propidium iodide-positive cells in MCF-7 treated with Evofos (1.5 µM) under normoxia/hypoxia, measured 72 h post-treatment. (**ii**) shows a bar graph of the percentage of dead MDA-MB-231 cells. (**B**) (**i**) shows bivariate analysis of Annexin-FITC/propidium iodide-positive MDA-MB-231 cells treated with Evofos (4 µM) under normoxia/hypoxia, measured 72 h post-treatment. (**ii**) shows a bar graph of the percentage of deaths in the case of MCF-7 cells. Data presented as mean ± SD; N = 3. *** *p* < 0.001 when compared to the respective treatment group; NS—not significant, as determined by one-way ANOVA followed by Bonferroni-adjusted post hoc test.

We, therefore, treated MCF-7 and MDA-MB-231 cells with 1.5 µM and 4 µM of Evofos, respectively, for all mechanistic studies described below. The Annexin-FITC/PI staining showed an increase in the percentage of PI-positive cells in both cell types under hypoxia compared to normoxia (Figure 2A,B). To ascertain the mechanisms of cell death, cells were harvested at the early time point of 48 h, and assays were conducted to detect apoptosis.

**Figure 3:**
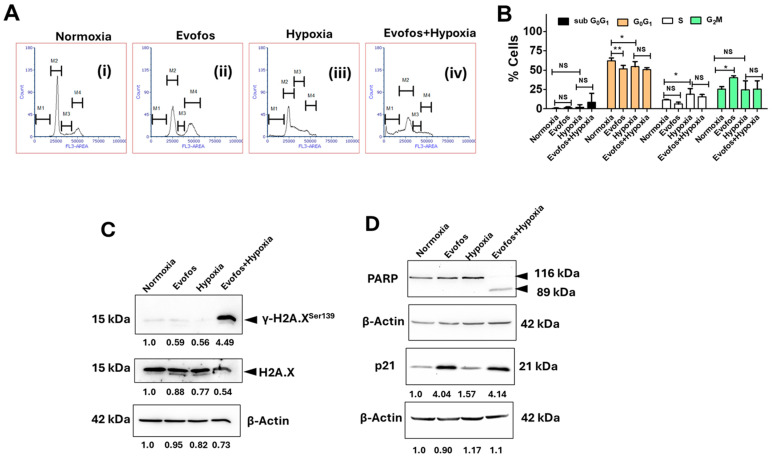
Conditioning MCF-7 cells with Evofos leads to alterations in the cell cycle and apoptotic cell death under hypoxia. (**A**) Representative flow cytometry histograms. (**B**) Bar graph showing the percentage of cells in different phases of the cell cycle—M1 = subG_0_G_1_; M2 = G_0_G_1_; M3 = S; and M4 = G_2_/M phase. Data presented as mean ± SD; N = 3. * *p* < 0.05, ** *p* < 0.01, NS = not significant when compared to respective treatment group as determined by one-way ANOVA followed by one-way ANOVA followed by Bonferroni-adjusted post hoc test. (**C**,**D**) Representative immunoblots showing changes in the expression of γ-H2A.X^Ser139^, PARP cleavage, and altered p21 levels as measured at 48 h after treatment. Values below the blots indicate the fold change in expression, normalized to the loading control.

**Figure 4:**
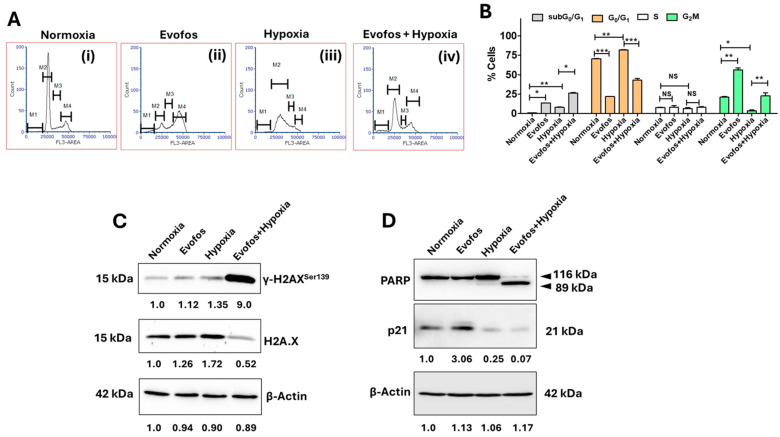
Conditioning MDA-MB-231 cells with Evofos leads to alterations in the cell cycle and induction of apoptotic cell death under hypoxic conditions. (**A**) Representative flow cytometry histograms. (**B**) Bar graph showing the percentage of cells in different phases of the cell cycle— M1 = subG_0_G_1_; M2 = G_0_G_1_; M3 = S; and M4 = G_2_/M phase. Data presented as mean ± SD; N = 3. * *p* < 0.05, ** *p* < 0.01, *** *p* < 0.001, NS = not significant when compared to respective treatment group as determined by one-way ANOVA followed by one-way ANOVA followed by Bonferroni-adjusted post hoc test. (**C**,**D**) Representative immunoblots showing changes in the expression of γ-H2A.X^Ser139^, PARP cleavage, and altered p21 levels as measured at 48 h after treatment. Values below the blots indicate the fold change in expression, normalized to the loading control.

Mechanistically, our results indicate that treatment with Evofos significantly enhanced G2/M cell cycle arrest under normoxic conditions in both cell lines. In contrast, cells exposed to hypoxia (1% O_2_) alone demonstrated an increase in G_0_/G_1_ cell cycle arrest. The combination of Evofos and hypoxia counteracted the G_0_/G_1_ arrest induced by hypoxia alone, resulting in a larger proportion of dying cells, as evidenced by an increase in sub-G_0_/G_1_ cells in both MCF-7 and MDA-MB-231 cell lines (Figure 3A,B and Figure 4A,B). Changes in cell cycle progression were also associated with the accumulation of DNA damage and alterations in p21 expression. Our Western blot analysis revealed a significant rise in phospho-H2A.X^Ser 139^ levels (a well-known biomarker for DNA double-strand breaks) in both MCF-7 and MDA-MB-231 cells treated with Evofos under hypoxic conditions (Figure 3C and Figure 4C). Furthermore, following Evofos treatment, MCF-7 cells showed increased p21 levels in both normoxia and hypoxia, whereas MDA-MB-231 cells exhibited a decrease in p21 levels under both hypoxia and Evofos + hypoxia treatments (Figure 3D and Figure 4D).

Furthermore, our results indicate an increase in the levels of cleaved PARP in MCF-7 and MDA-MB-231 cells conditioned with Evofos under hypoxic conditions compared to normoxic conditions (Figure 3D and Figure 4D). We also demonstrate the laddering of nucleosomal DNA, a classic indicator of apoptosis [26], along with caspase-3 activation in MDA-MB-231 cells and caspase-7 activation in MCF-7 cells (Supplementary Figure S2A,B). Taken together, these results suggest that Evofos conditioning under hypoxia is linked to apoptotic cell death.

### 3.2. Evofos-Mediated Cell Death Under Hypoxia Is Accompanied by Accumulation of ROS and Depleted Mitochondrial Membrane Potential

**Figure 5:**
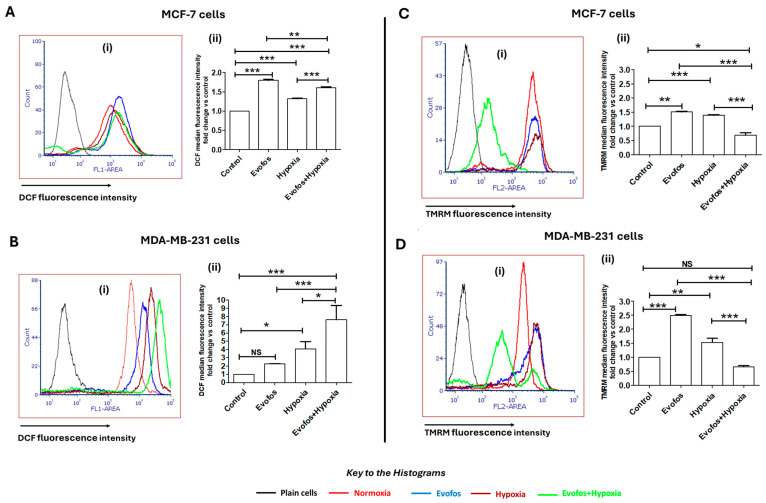
Evofos alters the intracellular ROS levels and mitochondrial membrane potential under both normoxic and hypoxic conditions. (**A**) (**i**) Histogram and (**ii**) bar graph showing changes in intracellular ROS in MCF-7 cells. (**B**) (**i**) Histogram and (**ii**) bar graph showing changes in intracellular ROS in MDA-MB-231 cells. (**C**) (**i**) Histogram and (**ii**) bar graph showing changes in mitochondrial membrane potential in MCF-7 cells. (**D**) (**i**) Histogram and (**ii**) bar graph showing changes in mitochondrial membrane potential in MDA-MB-231 cells. All measurements were done after 48 h of treatment. Data presented as mean ± SD; N = 3. * *p* < 0.05; ** *p* < 0.01; *** *p* < 0.001, NS = not significant when compared to the respective treatment groups as determined by one-way ANOVA followed by Bonferroni-adjusted post hoc test.

Changes in DCF fluorescence indicated the alteration in overall cellular ROS. MDA-MB-231 and MCF-7 cells treated with Evofos under normoxic conditions exhibited a 2.2-fold and 1.7-fold increase in overall cellular ROS, respectively, compared to the untreated control after 48 h of treatment (Figure 5A,B). Under hypoxic conditions, MDA-MB-231 cells demonstrated a 4-fold increase in DCF fluorescence, while MCF-7 cells showed a 1.3-fold increase. Conditioning the cells with Evofos intensified the cellular ROS levels under hypoxia, with MCF-7 cells displaying a 1.2-fold increase in DCF fluorescence, whereas MDA-MB-231 cells showed a 1.8-fold increase in DCF fluorescence compared to cells treated with hypoxia alone (Figure 5A,B).

Our data indicated that Evofos also influences mitochondrial membrane potential. MDA-MB-231 and MCF-7 cells showed a 2.4-fold and 1.5-fold increase in TMRM fluorescence, respectively, when treated with Evofos under normoxic conditions. Hence, Evofos led to hyperpolarization of mitochondria under normoxia. Conditioning the cells under hypoxia alone also had a similar impact on mitochondrial membrane potential, leading to hyperpolarization, with MDA-MB-231 and MCF-7 cells exhibiting a 1.5-fold and 1.3-fold increase in TMRM fluorescence intensity, respectively. In contrast, cells conditioned with Evofos under hypoxia showed a significant loss of mitochondrial membrane potential, as measured by a decrease in TMRM fluorescence intensity. Taken together, these results demonstrate that Evofos treatment under hypoxia causes oxidative stress, accompanied by a loss of mitochondrial potential (Figure 5C,D).

### 3.3. Evofos Prevents the Downregulation of cGAS-STING Levels and the Expression of Type I Interferon Signaling Genes Under Hypoxia

**Figure 6:**
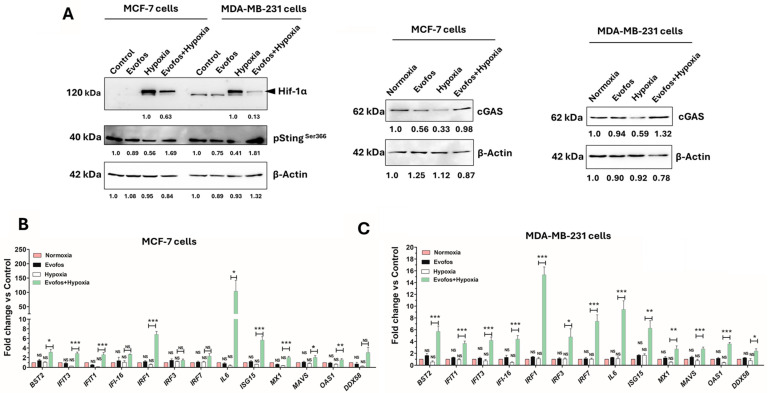
Conditioning cells with Evofos modified the expression of Hif-1α and cGAS/STING, as well as the gene expression of type I interferon. (**A**) The representative immunoblot analysis reveals alterations in Hif-1α, cGAS, and pSTINGSer366 levels following Evofos treatment in both normoxic and hypoxic conditions. Values below the blots indicate the fold change in expression, normalized to the loading control. Changes in the gene expression of type I interferon signaling genes in MCF-7 cells (**B**) and MDA-MB-231 cells (**C**) after Evofos treatment in normoxia or hypoxia, as measured 24 h after treatment. Data presented as mean ± SD; N = 3. * *p* < 0.05, ** *p* < 0.01, *** *p* < 0.001, NS = not significant when compared between respective treatment groups as measured by one-way ANOVA followed by Bonferroni-adjusted post hoc test.

We further investigated the changes in cGAS-STING levels in MCF-7 and MDA-MB-231 cells treated with Evofos under both normoxic and hypoxic conditions. Cytoplasmic DNA accumulation can occur due to either the release of damaged DNA fragments from the nucleus or depolarized mitochondria, which may subsequently activate the cGAS-STING pathway [27]. Our findings indicated that hypoxia-treated cells exhibited low levels of cGAS and a corresponding decrease in pSTING^Ser366^, as shown in Figure 6A for both MCF-7 and MDA-MB-231 cells after 24 h of treatment. Notably, Evofos treatment counteracted the suppressive effect of hypoxia on cGAS and pSTING^Ser366^ expression, resulting in increased levels compared to the hypoxia-only group in both cell lines. This increase was attributed to Evofos lowering Hif-1α levels (Figure 6A) in both tested cell lines, thereby promoting an immune response from the cells. This observation further supported the positive impact of Evofos on activating the cGAS/STING pathway.

We also evaluated the expression changes of genes related to the type I interferon pathway after Evofos treatment in both normoxic and hypoxic environments. The genes analyzed were *BST2*, *DDX58*, *IFIT1*, *IFIT3*, *IFI-16*, *IRF1*, *IRF3*, *IRF7*, *IL6*, *ISG15*, *MX1*, *MAVS*, and *OAS1*. These genes were selected based on a prior study that investigated the impact of hypoxia on the transcription and translation of components in the type I IFN signaling pathway [16]. These genes are primarily engaged in the innate immune response to viral infections; however, they are also implicated in tumor suppression. For instance, *IFIT1*, *IFIT3*, *IFI-16*, *ISG15*, *MX1*, and *OAS1* have been shown to have tumor-suppressive function [28].

We observed that hypoxia (1% O_2_) alone altered the expression of several of these genes, as indicated in Figure 6B,C. In both MDA-MB-231 and MCF-7 cells, hypoxia resulted in the downregulation of *BST2*, *DDX58*, *IFI-16*, *MX1*, and *OAS1* when compared to untreated cells, assessed 24 h post-treatment. Thus, conditioning with Evofos, followed by hypoxia treatment, significantly increased the expression of nearly all tested genes in both cell lines, suggesting that Evofos may enhance the anti-tumor immune response via the type I interferon pathway.

### 3.4. Evofos Conditioned Hypoxic Cells Are Sensitive to NK-92-Mediated Cytotoxicity

As indicated above, conditioning the cells with Evofos in hypoxic conditions restored the levels of cGAS/STING and the expression of type-I-interferon-related genes. Consequently, we investigated the sensitivity of MCF-7 and MDA-MB-231 cells to NK-92-mediated cytotoxicity. For this, the cells were conditioned with Evofos and incubated under normoxic or hypoxic conditions for 24 h. They were then stained with CellTracker Green dye and subsequently co-cultured with NK-92 cells for 4 h. The cells received Evofos treatment for 24 h since longer durations led to cell death from Evofos treatment itself, which would interfere with the analysis of co-culturing effects with NK-92 cells.

**Figure 7:**
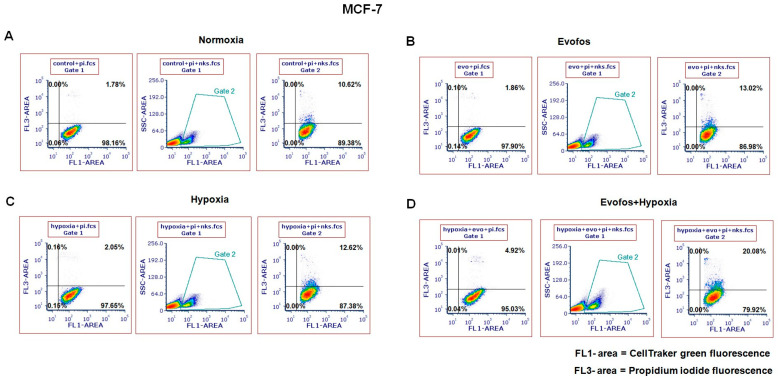
Evofos conditioning prepares MCF-7 cells for NK-92-mediated cytotoxicity. The representative dot plots illustrate the cytotoxic impact of NK-92 cells on Evofos-treated MCF-7 cells (for 24 h) in normoxia or hypoxia. Each treatment group was co-cultured with or without NK-92 cells and then stained with propidium iodide. MCF-7 cells undergoing death (lysis) are indicated by the upper right quadrant in the dot plots, which are positive for both CellTracker Green (FL-1; 488/525 nm) and propidium iodide uptake (FL3; fluorescence 488/630 nm). Panels (**A**) indicate untreated or control cells in normoxic conditions, (**B**) indicate Evofos-treated cells in normoxia, (**C**) indicate hypoxia-treated cells, and (**D**) indicate the cells treated with Evofos + Hypoxia. Each treatment group without NK-92 cells served as an internal control, indicative of the spontaneous death caused by Evofos or hypoxia alone. The data reflect results from at least three independent experiments. pi = propidium iodide; nks = NK-92 cells.

**Figure 8:**
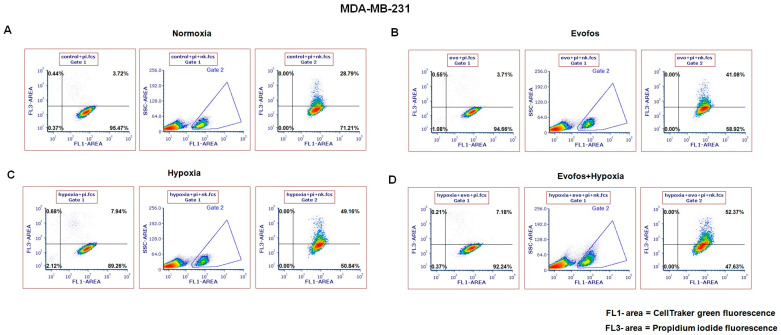
Evofos conditioning led to significant cell death in MDA-MB-231 cells when cultured in the presence of NK-92 cells. The representative dot plots illustrate the cytotoxic impact of NK-92 cells on Evofos-treated MDA-MB-231 cells (for 24 h) in normoxia or hypoxia. Each treatment group was co-cultured with or without NK-92 cells and then stained with propidium iodide. MDA-MB-231 cells undergoing death (lysis) are indicated by the upper right quadrant in the dot plots, which are positive for both CellTracker Green (FL-1; 488/525 nm) and propidium iodide uptake (FL3; fluorescence 488/630 nm). Panels (**A**) indicate untreated or control cells in normoxic conditions, (**B**) indicate Evofos-treated cells in normoxia, (**C**) indicate hypoxia-treated cells, and (**D**) indicate the cells treated with Evofos + Hypoxia. Each treatment group without NK-92 cells served as an internal control, indicative of the spontaneous death caused by Evofos or hypoxia alone. The data reflect results from at least three independent experiments. pi = propidium iodide; nks = NK-92 cells.3.5. Tumor Hypoxia Correlates with STING Downregulation in Breast Cancer Cohorts.

The flow cytometry results revealed that Evofos treatment increased sensitivity in both MCF-7 and MDA-MB-231 cells. Figure 7 illustrates the cytotoxic impact of NK-92 cells on Evofos-conditioned MCF-7 cells in both normoxic and hypoxic settings. Untreated MCF-7 cells co-cultured with NK-92 cells exhibited a lysis rate of 10.62% (Figure 7A), whereas Evofos-conditioned MCF-7 cells in normoxia showed a lysis rate of 13.02% (Figure 7B). Additionally, MCF-7 cells co-cultured with NK-92 under hypoxia exhibited 12.62% lysis (Figure 7C), whereas the combination of Evofos and hypoxia increased lysis to 20.08% (Figure 7D). In contrast, MDA-MB-231 cells demonstrated a more robust response to NK-92-mediated cytotoxicity. MDA-MB-231 cells, being easily targeted by NK-92 cells, exhibited a 28.79% cell lysis rate when untreated and co-cultured with NK-92 cells (Figure 8A). Cells conditioned with Evofos alone in normoxia showed a significant reduction in lysis at 41.08% (Figure 8B), while hypoxia alone resulted in 49.16% cell lysis when co-cultured with NK-92 cells. Evofos treatment in a hypoxic environment resulted in 52.37% lysis of MDA-MB-231 cells during co-culture with NK-92 (Figure 8D).

Together, these results suggest that cells conditioned with Evofos under hypoxic conditions display increased sensitivity to NK-cell-mediated cytotoxicity.

To assess the potential link between hypoxia, immune infiltration, and STING signaling, we explored gene expression data from the primary tumors of the TCGA breast cancer cohort (BRCA TCGA PanCancer). First, we evaluated the effect of hypoxia score (HS) and immune score (IS) alone on survival in breast cancer cohorts. We found that hypoxia was associated with worse survival in patients (Supplementary Figure S3A). In this context, HS high tumors exhibited significantly reduced expression of STING (*p* < 0.005) compared to HS low tumors (Supplementary Figure S3B). While the immune signature was linked to enhanced survival (Supplementary Figure S3C), we observed that STING levels were significantly higher in IS high tumors compared to IS low tumors (*p* < 0.005) (Supplementary Figure S3D).

**Figure 9:**
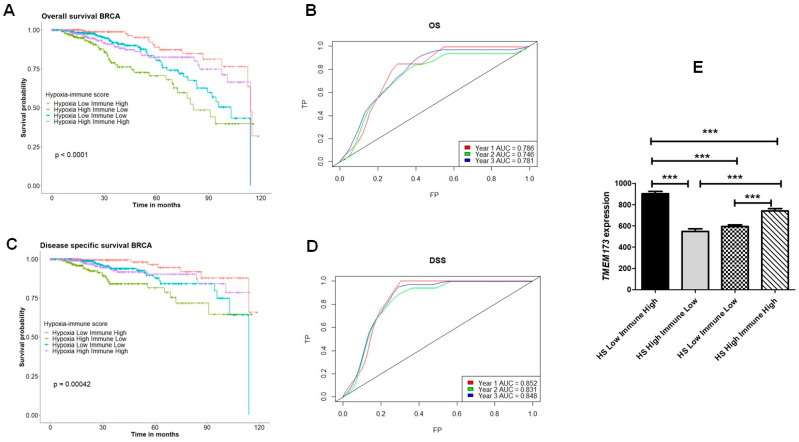
The hypoxia and immune-score-based classification of TCGA breast cancer cohorts demonstrates significant variation in STING expression among the groups. Kaplan–Meier survival plots compare the four groups: Hypoxia Low/Immune Low, Hypoxia Low/Immune High, Hypoxia High/Immune Low, and Hypoxia High/Immune High (**A**,**C**). ROC curves depict the predictive efficiency of the hypoxia signature for overall survival (OS) (**B**) and disease-specific survival (DSS) (**D**), considering the 1-, 2-, and 3-year survival rates compared between the HS Low Immune High and HS High Immune Low groups. AUC: area under the curve; FP: false positive (1—specificity); TP: true positive (sensitivity). (**E**) Expression of the TMEM173 transcript (STING) across the four groups (*** *p*-values < 0.001 when compared to respective groups). The Log-rank test was employed to evaluate the null hypothesis of equality of survival distributions, while comparison of the expression of the *TMEM173* across groups was determined by one-way ANOVA, followed by Bonferroni-adjusted post hoc test.

Thus, there is a correlation between hypoxia and reduced expression of STING, and this reduction could also be linked to a low immune score in the TCGA breast cancer cohort. To further determine whether STING expression varies in tumors classified by their degree of hypoxia and immune inflammation, patients were categorized into four groups: Hypoxia High/Immune Low, Hypoxia High/Immune High, Hypoxia Low/Immune High, and Hypoxia Low/Immune Low, based on both their HS and IS. Our analysis indicated a significant association with overall survival (OS) (Figure 9A) and disease-specific survival (DSS) (Figure 9C), with the most considerable differences observed between the Hypoxia Low/Immune High and Hypoxia High/Immune Low groups. Indeed, the predictive value for the combined signature, as determined by ROC curve analysis, revealed an AUC of >0.7 for predicting years 1, 3, and 5 OS (Figure 9B) and DSS (Figure 9D). In this context, Hypoxia Low/Immune High tumors, which exhibited the best overall and disease-specific survival probability, had the highest STING expression (Figure 9E). In contrast, the lowest expression of STING was found in the Hypoxia High/Immune Low group (Figure 9E), which also demonstrated the worst overall and disease-specific survival probability. These findings suggest that tumors with high hypoxia scores can impact immune infiltration and that hypoxia is associated with STING expression.

## 4. Discussion

**Figure 10:**
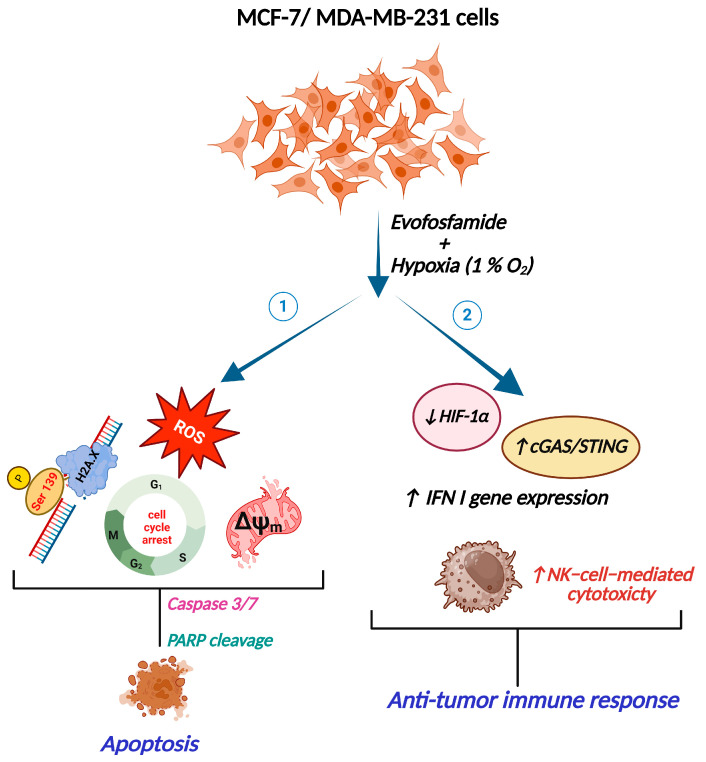
The graphical abstract illustrates the impact of Evofos on MCF-7 and MDA-MB-231 cells under hypoxia (1% O_2_). Evofos exerted a two-pronged effect in both MCF-7 and MDA-MB-231 cells under hypoxic conditions: (**1**) Evofos, being a HAP, induced DNA double-strand breaks, oxidative stress, and altered mitochondrial membrane potential (ΔΨ_m_), leading to apoptosis. (**2**) On the other hand, Evofos reduced HIF-1α levels and alleviated type I IFN gene expression and NK-92-cell-mediated cytotoxicity, which can lead to an anti-tumor immune response. The graphical abstract has been created using BioRender.com.

Evofos is a second-generation HAP whose activity within the cell is inversely proportional to O_2_ tension [29], resulting in cellular death through extensive DNA damage and the activation of associated pathways. The present study demonstrated that Evofos, under hypoxia (1% O_2_), induced cell death in two distinct breast cancer cell lines, MCF-7 and MDA-MB-231. These findings suggest the efficacy of Evofos against both hormone-receptor-positive and hormone-receptor-negative breast cancer. Our findings align with a previous study that demonstrated a similar cell-killing effect of Evofos on a high-grade glioma cell model under hypoxic conditions [30]. Treating MCF-7 and MDA-MB-231 cells with Evofos in a hypoxic environment led to DNA damage, oxidative stress, and changes in mitochondrial membrane potential, culminating in apoptotic cell death. Furthermore, Evofos promoted the overexpression of genes related to the type I IFN pathway and heightened NK-92-cell-mediated cytotoxicity in both breast cancer cell lines tested. This indicates Evofos’s immunomodulatory activity against breast cancer cells under hypoxic conditions (Figure 10).

We conducted all hypoxia-related experiments at a clinically relevant oxygen percentage (1%) as earlier reports indicate that intra-tumoral oxygen concentrations in breast cancer are approximately 1–1.5% O_2_ [31,32]. Several schools of thought suggest that Evofos is activated under very low O_2_ tensions; however, a seminal study by Hunter and colleagues [33] demonstrated that Evofos can be partially activated by mitochondrial electron transport chain activity, which explains its biological activity under normoxia. Our findings, consistent with an earlier report, indicate that Evofos prevents cell cycle progression by inducing G_2_/M arrest [9]. The one-electron reduction of Evofos is a reversible reaction when molecular O_2_ is present. Hence, under normoxic conditions, Evofos is typically kept in the non-toxic pro-form [34]. On the contrary, the changes in cellular parameters in Evofos-treated cells during normoxia observed in the present findings may stem from two factors. Firstly, a small proportion of the intermediate radicals may escape and fragment to produce bromo-iso-phosphoramide mustard (Supplementary Figure S4). Secondly, the Evofos molecules may be transformed into bromo-iso-phosphoramide mustard due to mitochondrial activity [33]. Conversely, under hypoxia, all Evofos molecules would be converted entirely to bromo-iso-phosphoramide mustard, resulting in profound cellular damage.

Data obtained from the viability experiment indicated that Evofos conditioning led to a significant decline in cell viability under hypoxia, associated with oxidative stress and the induction of DNA damage in both cell lines, MCF-7 and MDA-MB-231, compared to normoxia. The oxidative stress induced by Evofos is attributed to its ability to inactivate the thioredoxin reductase and glutathione reductase enzymes, thereby lowering intracellular reduced glutathione (GSH) levels, which are crucial for maintaining intracellular redox homeostasis and detoxification [35]. Conversely, hypoxia-associated ROS could be attributed to acidification [36] and impairment of mitochondrial electron transport via the cytochrome chain. This can further lead to decreased ATP levels and ROS accumulation, resulting in reduced intracellular antioxidant levels [37,38].

In addition to the redox imbalance, our data indicated that Evofos under normoxic conditions resulted in hyperpolarized mitochondria. Treating cells with hypoxia alone also led to this phenomenon. Conversely, combining Evofos with hypoxia significantly decreased mitochondrial membrane potential in both MCF-7 and MDA-MB-231 cells. The combination of Evofos and hypoxia produced an additive effect on the antioxidant system and membrane potential as both cell lines exhibited increased levels of ROS and decreased mitochondrial membrane potential. Hyperpolarization of mitochondrial membranes occurs due to impaired electron transport chain (ETC) and ATP synthase activity, resulting in an excessive buildup of electrons, or it can also arise from an excessive accumulation of calcium ions [39,40]. Hypoxic conditions impair ATP production via the mitochondrial ETC, a significant factor contributing to the hyperpolarized mitochondria observed in our results [39,41]. The exact mechanism by which Evofos treatment under normoxia causes the hyperpolarization of mitochondrial membranes is unknown; we presume it may be an indirect effect. Currently, we hypothesize that some Evofos molecules may be converted into their active form (as shown in Supplementary Figure S4) due to electron transport chain activity or other oxidation–reduction reactions that could lead to mitochondrial or nuclear DNA damage. An intact mitochondrial genome is essential for the proper stoichiometric assembly of the electron transport chain protein complexes as it encodes several proteins that are part of these complexes in addition to proteins encoded by nuclear DNA [42]. Therefore, treating cells with Evofos under normoxia may induce stoichiometric disruption of protein complexes in the mitochondrial electron transport chain (ETC) due to mitochondrial DNA damage, which could be the cause of hyperpolarized mitochondria. Although it is currently unknown, Evofos may directly inhibit mitochondria-localized antioxidants, such as thioredoxin reductase and glutathione reductase enzymes. This inhibition can also contribute to oxidative-stress-related mitochondrial impairment [43], leading to hyperpolarized mitochondria under normoxic conditions.

Along with elevated ROS and a decline in mitochondrial membrane potential, Evofos conditioning under hypoxia resulted in DNA double-strand breaks, altered cell cycle progression, and apoptosis. While Evofos induced a G_2_/M arrest under normoxia, accompanied by an elevation in p21, when compared to the control in both cell lines, we did not observe the accumulation of phospho-H2A.X^Ser139^ by Western blot analysis, suggesting that Evofos did not induce considerable DNA damage at this concentration in normoxia. Conversely, a significant increase in phospho-H2A.X^Ser139^ was observed in Evofos + hypoxia-treated cells, accompanied by a reduction in G_2_/M arrest and an increase in the subG_0_G_1_ population in both MCF-7 and MDA-MB-231 cells. Apoptotic cell death was evidenced by the presence of DNA ladder formation and PARP cleavage in both cell lines. Since MCF-7 lacks a functional caspase 3 [44], apoptosis in this cell line may be driven by the release of endonuclease G from depolarized mitochondria, leading to the fragmentation of nucleosomal DNA [45] and the activation of caspase 7, which causes the apoptotic cleavage of PARP [46]. In summary, Evofos-induced cell death under hypoxia is linked to redox imbalance, mitochondrial dysfunction, and DNA damage that results in apoptosis.

The success of chemotherapy, radiotherapy, and immunotherapy relies on a robust activation of type I interferons in the tumor microenvironment (TME). Type I interferons play a significant role in immunosurveillance mechanisms by promoting the infiltration of cytotoxic T and NK cells and controlling neoplastic progression [47]. It has been reported that 50% of BC are hypoxic, which further causes therapy-related resistance [48]. Furthermore, hypoxia-induced immune evasion occurs through the shedding of major histocompatibility complexes, the upregulation of PD-L1 on cancer cells and myeloid-derived suppressor cells, and the dampening of cytosolic DNA sensing pathways due to epigenetic changes [15,49,50,51]. A recent study using breast cancer models demonstrated that hypoxic cancer cells silence type I IFN signaling to acquire immune evasion [16]. Given that hypoxia is a dynamic and continuously fluctuating phenomenon within the TME, it can significantly hinder type I IFN production and facilitate immune evasion.

Hypoxia signatures have emerged as a method to illustrate the hypoxic state of a tumor based on the expression levels of hypoxia-related genes. These signatures serve as indicators of tumor hypoxia, enabling the comparison of survival rates and molecular events among tumors based on their hypoxia levels [24]. An increasing number of such signatures have become available in the literature; however, the most robust one remains the Buffa signature [21]. Sorting the breast cancer TCGA cohort based on the hypoxia score from this signature indicated that tumors with high hypoxia had lower survival rates and were associated with reduced levels of STING transcripts. Furthermore, to simultaneously assess the impact of the immune microenvironment, a tissue-agnostic 18-gene Expanded Immune Signature was applied to indicate the immune response of the tumors through an immune score [22]. The classification of patients based on their immune and hypoxia scores demonstrated that both hypoxia levels and immunity significantly impacted overall survival and improved prognosis prediction. The evaluation of STING transcript levels among the groups showed that tumors in patients classified as having high hypoxia but low immunity were linked to suppression of STING transcripts. Findings from the BC cohort suggest that tumor hypoxia is related to STING downregulation.

Our findings align with prior reports [16], demonstrating a downregulation of the cGAS-STING axis under hypoxic conditions in both MCF-7 and MDA-MB-231 cell lines. Conversely, Evofos conditioning countered the suppressive effects of hypoxia and increased the expression of type I interferon signaling genes while restoring the cGAS/STING signaling pathway. The restoration of the cGAS/STING pathway may also be attributed to the genotoxic potential of Evofos, resulting in the accumulation of DNA in the cytosol, which can originate from either the nucleus or depolarized mitochondria [52]. cGAS-STING activation in the TME significantly influences tumor immunogenicity. STING-deficient mice do not achieve optimal NK cell cytotoxicity, highlighting the importance of STING activation for NK-cell-based tumor rejection [53]. Conversely, increased cGAS-STING activity in the TME is associated with heightened infiltration of NK cells and other immune cells [54]. Additionally, hypoxic stress induces functional suppression of NK cells in the TME, while alleviation of hypoxia plays a crucial role in restoring NK cell function [54,55]. Coinciding with these facts, our results show that Evofos conditioning under hypoxia enhanced the cytotoxic activity of NK-92 cells compared to Evofos treatment in normoxia in both MCF-7 and MDA-MB-231 cells. NK-92 cells serve as an essential surrogate model for natural NK cells in cancer immunotherapy research; however, certain limitations exist. It is important to note that the in vitro NK-92-cell-mediated cytotoxicity assay evaluates the short-term cytolytic capacity of the cells. Its long-term efficacy against Evofos-treated MCF-7/MDA-MB-231 cells warrants evaluation using in vivo xenograft or orthotopic models in either beige, NSG/NOG, or NOD-SCID mice [56]. NK-92 cells depend on IL-2 levels and lack several surface receptors. Notably, they are missing most of the inhibitory killer cell immunoglobulin-like receptors and exhibit low levels of KIR2DL4 [57,58]. Nonetheless, NK-92 cells have demonstrated exceptional cytotoxicity against various tumor types in preclinical studies. Clinical trials have confirmed the safety of NK-92 infusions, even at elevated doses. Additionally, to combat tumor resistance and enhance targeted therapy, NK-92 could be modified to express various chimeric antigen receptors, making it very promising for the development of effective treatments [59]. This aligns with prior research indicating that targeting hypoxia via Evofos improves the effectiveness of immune checkpoint blockade by enhancing cytotoxic T-cell activity in prostate cancer [14]. These observations suggest that Evofos has the potential to act as a STING agonist, counteracting hypoxia associated with the downregulation of type I interferon and enhancing NK cell cytotoxicity in BC cells. Type I interferons regulate the homeostasis, maturation, and activation of NK cells within the TME, thereby contributing to the antitumor immune response [53].

The data obtained from two Phase III randomized clinical trials—MAESTRO (Evofos-340 mg/m^2^ + gemcitabine-1000 mg/m^2^ or placebo + gemcitabine against locally advanced, unresectable, or metastatic PDAC) and SARC021 (Evofos-300 mg/m^2^ + Doxorubicin-75 mg/m^2^ or placebo + doxorubicin against advanced, unresectable, metastatic intermediate, or high-grade soft sarcoma)—indicate that patients treated with Evofos in combination with gemcitabine/doxorubicin experienced a range of adverse effects and a decrease in overall survival rates compared to those treated with gemcitabine/doxorubicin alone [12,13]. Despite the adverse effects reported in these clinical trials, we still maintain that Evofos could benefit patients selected based on their tumor’s hypoxic state [4,5,60]. Currently, conventional drugs such as paclitaxel and cisplatin are associated with adverse effects in patients, including skin reactions, mucositis, hematologic alterations, and organ dysfunction [61,62,63]. It is expected that Evofos alone would inflict less normal tissue toxicity compared to conventional drugs, as it requires a hypoxic environment for its complete activation. This is further supported by previous findings wherein Evofos, in combination with radiotherapy (X-rays), did not induce normal tissue toxicity in the Nu-Foxn1-nu (NU/NU) mouse model for esophageal cancer [8]. The severe side effects of Evofos reported in the two Phase III clinical trials may be due to the additive effect of the combination treatment with Evofos along with gemcitabine in MAESTRO or doxorubicin in SARC021, as mentioned above. Our current findings demonstrate that, under normoxic conditions, Evofos alters cellular parameters, including the elevation of cellular ROS, altered mitochondrial membrane potential, and a G2/M arrest. Therefore, it is reasonable to believe that a combination of Evofos and any conventional chemotherapy drug could lead to significant cellular damage as Evofos may act as a sensitizer.

The toxicity of Evofos to normal tissue can be reduced by using targeted delivery strategies, such as formulations or nanoparticles, to deliver the drug directly to tumor sites. This approach accurately delivers Evofos to tumor cells, allowing for lower dosages and thereby minimizing adverse effects [64]. This approach can also be paired with immunotherapy using anti-CTLA4 or anti-PD1 agents and tested against both TNBC and non-TNBC subsets of BC. Furthermore, our current findings and others [9,65] have demonstrated that Evofos reduces the levels of HIF-1α. So, it can be anticipated that Evofos treatment may further decrease HIF-1α-controlled pathways related to metabolism, including glycolysis, lipid synthesis, and amino acid metabolism, which are crucial for the metabolic plasticity and progression of hypoxic tumors, such as TNBC [66]. Earlier reports also suggest that Evofos can improve oxygenation [34]. Thus, Evofos can potentially enhance the effectiveness of immunotherapy by upregulating type I IFN signaling and reducing hypoxia pockets within the TME [60,67]. Additionally, the efficacy of Evofos-based therapy can be enhanced by stratifying patients based on the severity of hypoxia [68]. Therefore, these properties of Evofos collectively emphasize its clinical significance and indicate that it should be evaluated as a promising anti-cancer agent for targeting hypoxic tumors, including breast cancer (BC).

## 5. Conclusions

In summary, our findings suggest that Evofos can effectively target breast cancer by stimulating anti-tumor immune responses and inducing apoptosis. Moreover, our findings demonstrate that Evofos is effective against both triple-negative breast cancer (TNBC) and non-TNBC cells. Additionally, Evofos may convert “immune-cold” hypoxic tumors into “immune-hot” ones by activating STING signaling, potentially improving treatment outcomes in tumors resistant to immune checkpoint blockade, which warrants further investigation.

## Data Availability

The data will be made available upon reasonable request.

## Supplementary Materials

The following supporting information can be downloaded at https://www.mdpi.com/article/10.3390/cancers17121988/s1. Table S1: List of antibodies used in the study. Table S2: List of primers used for the real-time PCR experiment. Figure S1: Dot plots representing flow cytometry analysis for the cytotoxic potential of NK-92 cells (effector cells) against K562 cells (target cells). Figure S2: MCF 7 cells (A) and MDA-MB-231 cells (B) treated with Evofos under hypoxia exhibit apoptotic cell death, as confirmed by DNA fragmentation assay and immunoblot analysis for caspase 3/7. Figure S3: Independent sorting of TCGA breast cancer cohorts based on Hypoxia Score (HS) and Immune Score (IS). (A) Kaplan–Meier survival plots compare overall survival between HS High and HS Low. (B) Increased hypoxia score is associated with decreased levels of the TMEM173 transcripts. (C) Kaplan–Meier survival plots compare overall survival between IS High and IS Low. (D) A decreased immune score is associated with lower levels of the TMEM173 transcripts. ** signifies *p*-value < 0.0021; and *** signifies *p*-value < 0.0001. Figure S4: Schematics for intracellular conversion of Evofosfamide under normoxia and hypoxia. The image is adapted from Kishimoto et al., 2021 [34]. Briefly, Evofosfamide (A) is converted to a radical (B) in the presence of 1-electron reductases, which can either break down to produce bromo-isophosphoramide mustard (Br-IPM; D) under hypoxia that alkylates with DNA, as shown in E. Under normoxia, it is believed to convert back to the original prodrug due to the presence of molecular oxygen (C). We envision that the conversion of the radical back to the pro-form of the drug (step B to C) may be altered due to mitochondrial activity.

## Abbreviations

The following abbreviations are used in this manuscript:

BC	Breast cancer
cGAS	Cyclic GMP–AMP synthase
CTLA4	Cytotoxic T-lymphocyte-associated protein 4
DCFDA	2′,7′-Dichlorofluorescin diacetate
DSS	Disease-specific survival
ETC	Electron transport chain
Evofos	Evofosfamide
HAP	Hypoxia-associated prodrug
HS	Hypoxia score
IFN	Interferon
IL-2	Interleukin 2
IS	Immune score
KIR2DL4	Killer Cell Immunoglobulin-Like Receptor, Two Ig Domains and Long Cytoplasmic Tail 4
OS	Overall survival
PCR	Polymerase chain reaction
PD1	Programmed death-1
ROS	Reactive oxygen species
STING	Stimulator of Interferon Genes
TME	Tumor micro-environment
TMRM	Tetramethylrhodamine, methyl ester
