# Supplementary material for: Evofosfamide Enhances Sensitivity of Breast Cancer Cells to Apoptosis and Natural-Killer-Cell-Mediated Cytotoxicity Under Hypoxic Conditions
Source: Cancers (Basel). 2025 Jun 14;17(12):1988. doi: 10.3390/cancers17121988 (PMC12191244; doi:10.3390/cancers17121988)

RAW IMAGES OF THE WESTERN BLOTS IN MAIN AND SUPPLEMNTARY FIGUES

Title- Evofosfamide Enhances Sensitivity of Breast Cancer Cells to Apoptosis and Natural-Killer-Cell-Mediated Cytotoxicity Under Hypoxic Conditions

Shubhankar Das [1], Goutham Hassan Venkatesh [1], Walid Shaaban Moustafa Elsayed [2], Raefa Abou Khouzam [1], Ayda Shah Mahmood [1], Husam Hussein Nawafleh [1], Nagwa Ahmed Zeinelabdin [1], Rania Faouzi Zaarour [1], Salem Chouaib [1,3] *

[1] Thumbay Research Institute for Precision Medicine, Gulf Medical University, Ajman 4184, UAE

[2] College of Dentistry, Gulf Medical University, Ajman 4184, UAE

[3] INSERM UMR 1186, Integrative Tumor Immunology and Immunotherapy, Gustave Roussy, University Paris-Saclay, 94805 Villejuif, France

*Correspondence : salem.chouaib@gmu.ac.ae or sa-lem.chouaib@gustaveroussy.fr ;

Tel.: +971-6-7431333; Fax: +971-6-7431222

Figure 3C- MCF-7 (γ-H2A.X ^{Ser139}, H2A.X, β-Actin)

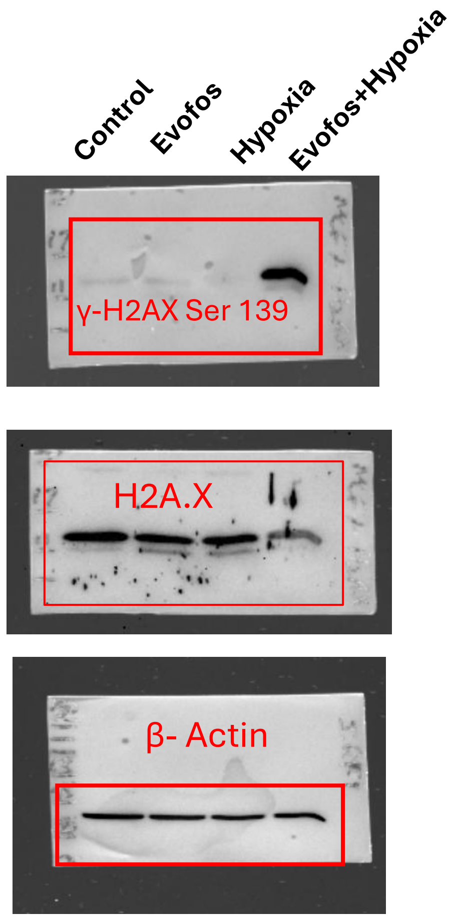

Figure 3D- MCF-7 (p21, PARP, β-Actin)

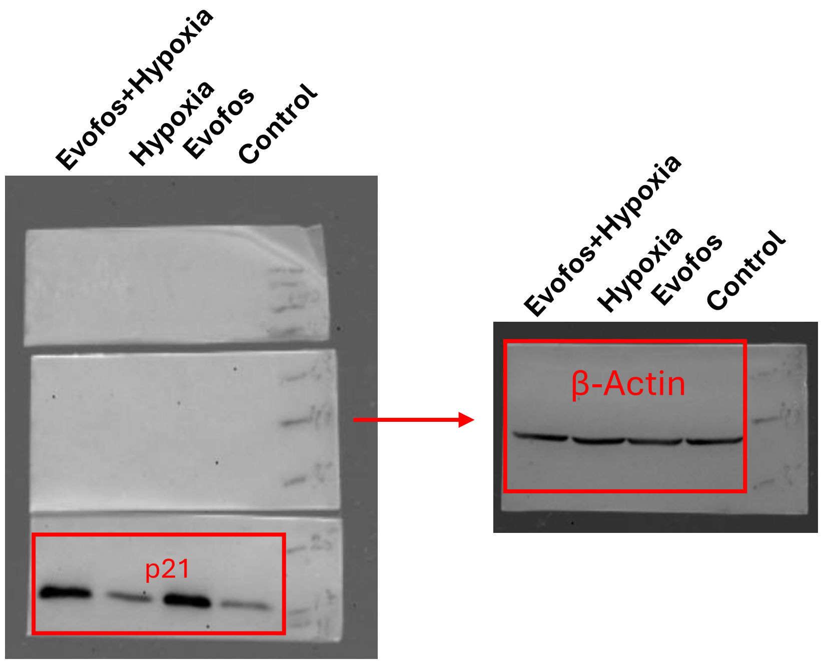

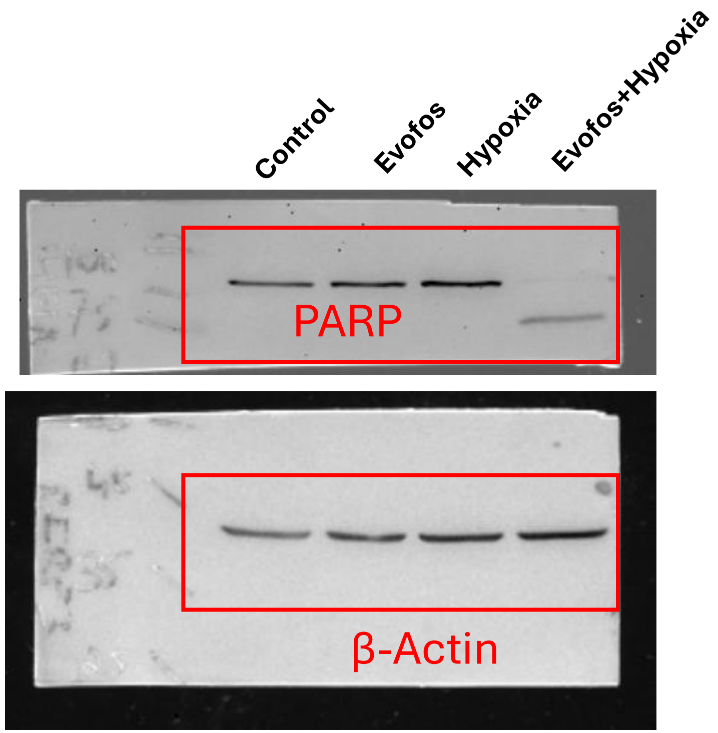

For Figure 3D, indicating changes in p21/actin in MCF-7 cells, the gel got inadvertently flipped while it was being assembled with the nitrocellulose membrane electro-blot was being set-up.

Figure 4C- MDA-MB-231 (γ-H2A.X ^{Ser139}, H2A.X, β-Actin)

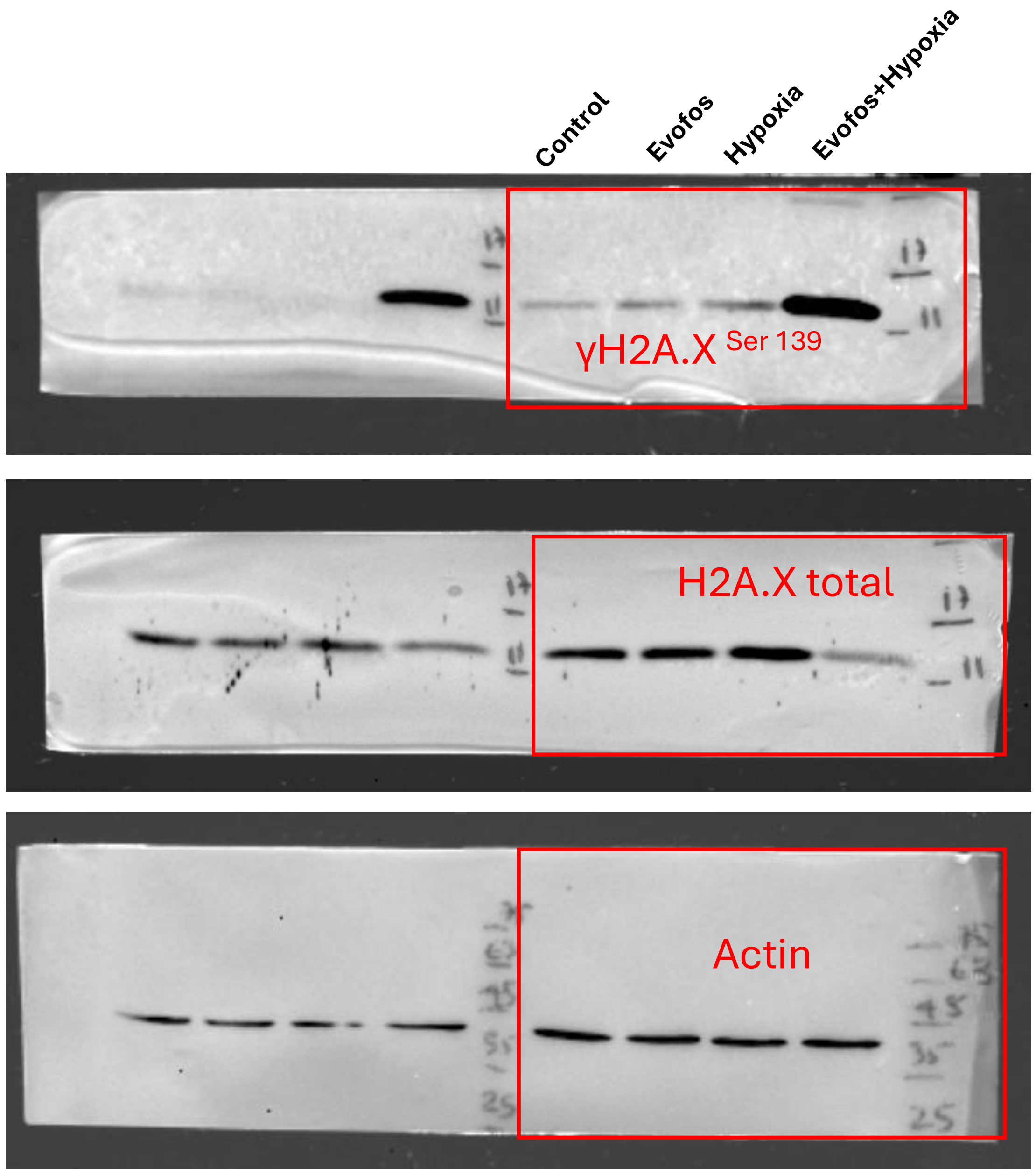

Figure 4D- MDA-MB-231 (PARP, p21, β-Actin)

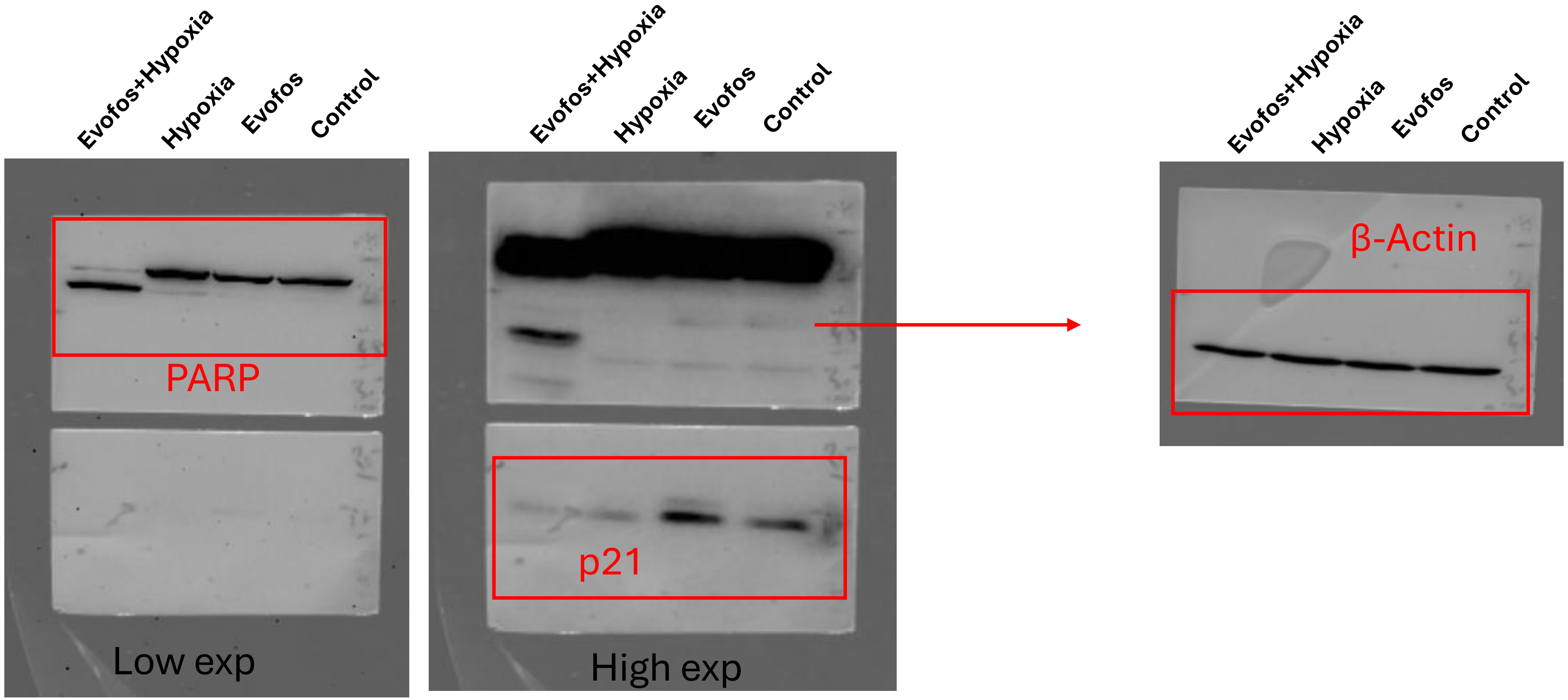

For Figure 4D, indicating changes in p21/actin in MDAMB231 cells, the gel got inadvertently flipped while it was being assembled with the nitrocellulose membrane electro-blot was being set-up.

Figure 6A- cGAS, β-Actin (MCF-7/ MDAMB231)

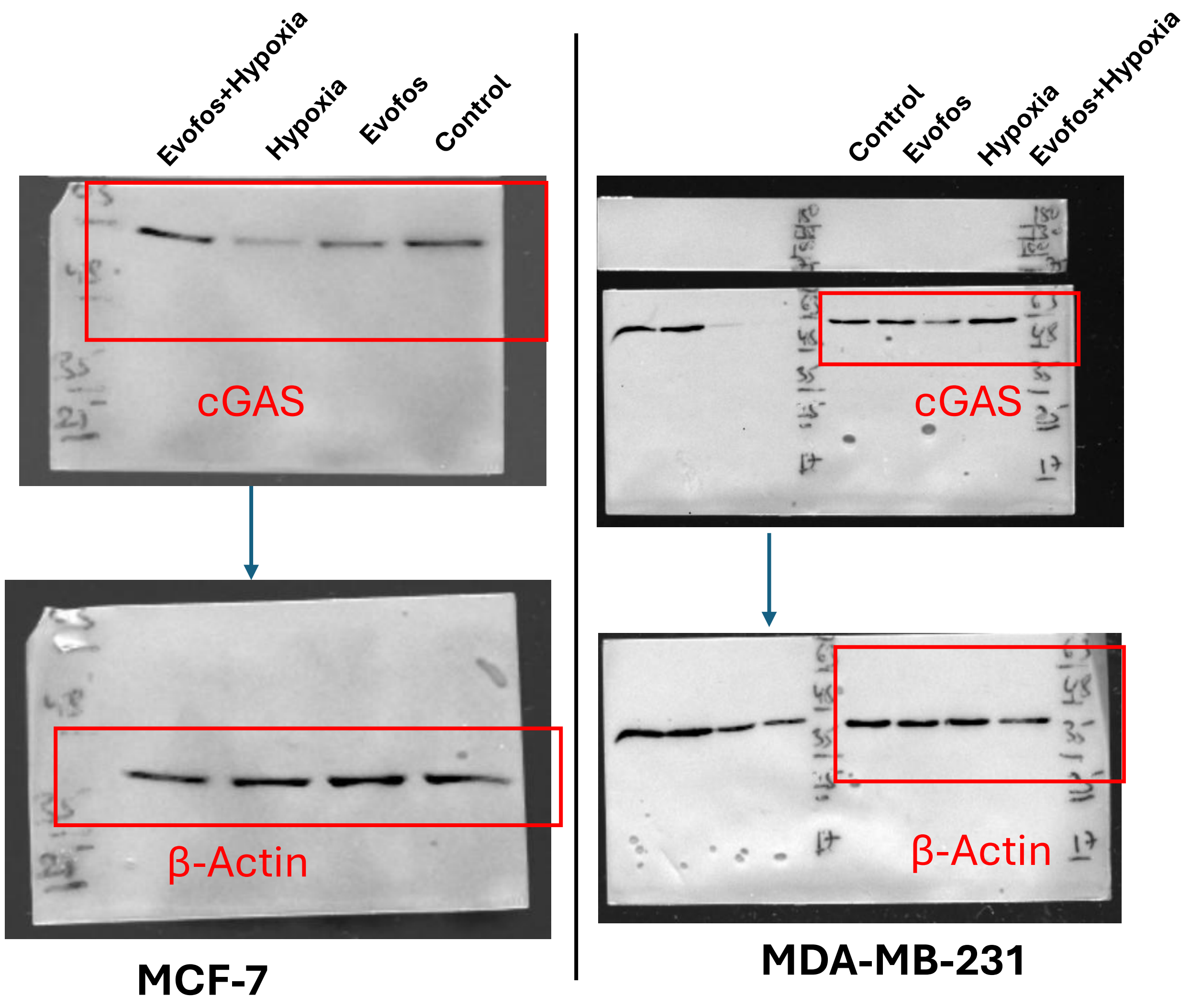

For Figure 6A, indicating changes in cGAS/actin in Mcf7 cells, the gel got inadvertently flipped while it was being assembled with the nitrocellulose membrane electro-blot was being set-

Figure 6A- HIF1α, β-Actin, pSTING^{Ser366} (MCF-7/ MDAMB231)

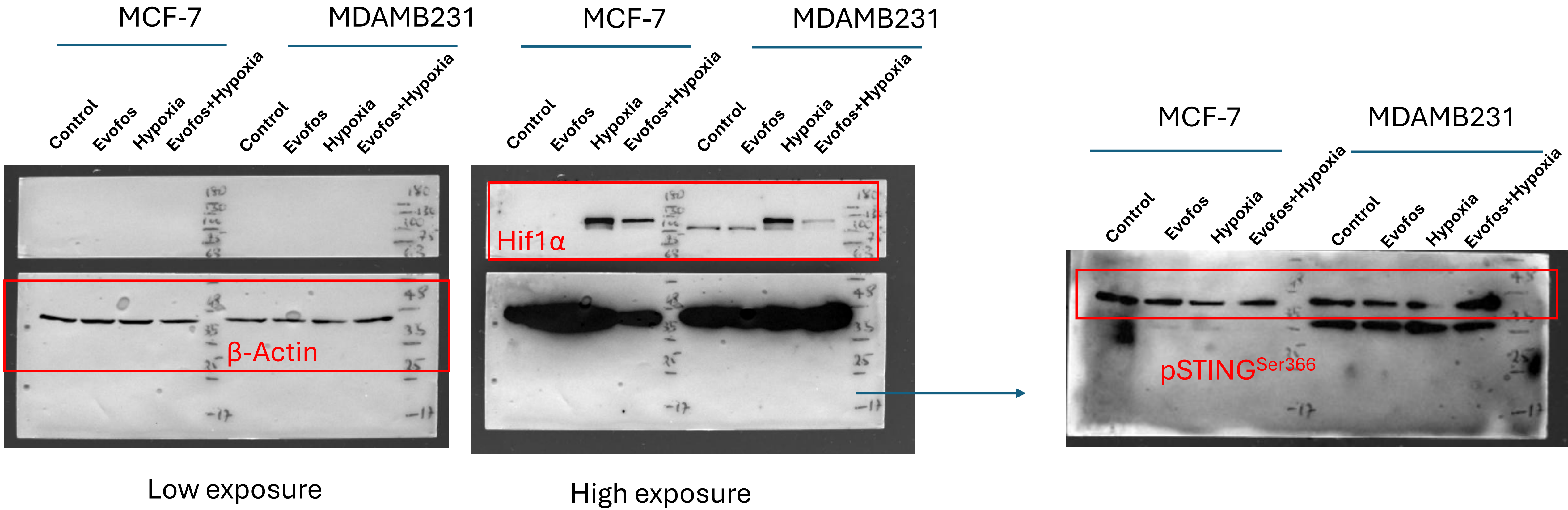

Supplementary Figure S2A- MCF7- cleaved caspase 7/ β-actin

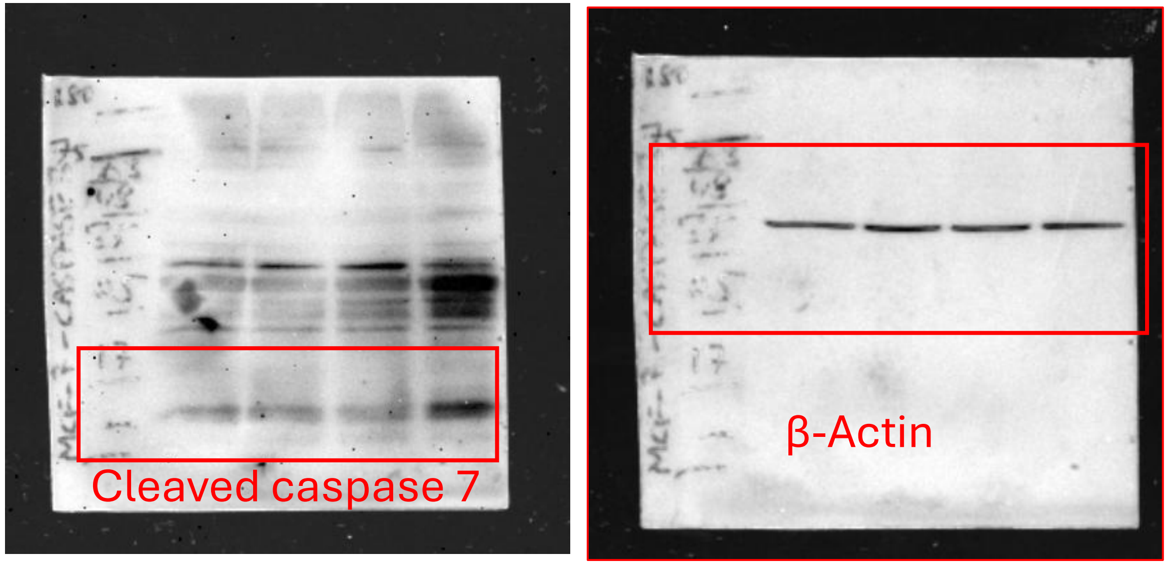

Supplementary Figure S3 B- MDAMB231- Caspase 3/ β-actin

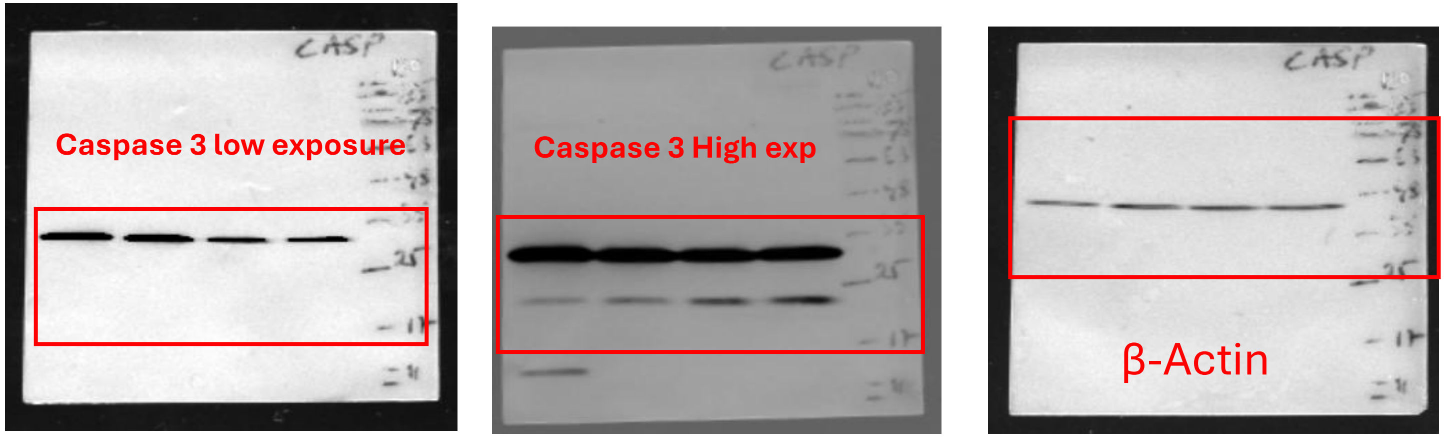

Supplement: Supplementary file 1 [file cancers-17-01988-s001.zip › Supplemenatry material II- raw blots.pdf]
