# Supplementary material for: Evofosfamide Enhances Sensitivity of Breast Cancer Cells to Apoptosis and Natural-Killer-Cell-Mediated Cytotoxicity Under Hypoxic Conditions
Source: Cancers (Basel). 2025 Jun 14;17(12):1988. doi: 10.3390/cancers17121988 (PMC12191244; doi:10.3390/cancers17121988)
Supplement: Supplementary file 1 [file cancers-17-01988-s001.zip › Supplementary material I- tables supplementary figures.pdf]

Supplementary information

Supplementary Table S1: List of antibodies used in the study

Name of antibody	Catalogue number/Make	Dilution
Anti- HIF1α – rabbit mAb	#14179 / Cell Signaling Technology	1:1500
Anti- Caspase 3 – rabbit mAb	#9662/ Cell Signaling Technology	1:6000
Anti- Caspase 7 – rabbit mAb	#9492/ Cell Signaling Technology	1:6000
Anti- PARP – rabbit mAb	#9542/ Cell Signaling Technology	1:6000
Human-Reactive STING Pathway Antibody Sampler Kit – rabbit mAb	#38866/ Cell Signaling Technology	1:1500
p21 rabbit mAb	#2947 / Cell Signaling Technology	1:5000
Anti- β-Actin – mouse mAb	sc-47778 / Santa Cruz Biotechnology	1:8000
Anti- Histone H2A.X- rabbit	#2595/ Cell Signaling Technology	1:5000
Anti- phosphor- H2A.X$^{Ser\ 139}$ – mouse mAb	05-636-I / Sigma Aldrich	1:4000
Goat anti-rabbit IgG (H+L) -HRP conjugated	# 31460/ ThermoFisher Scientific	1:12000
Anti- CD3 Mouse mAb	#86774 / CD3 (UCHT1) (FITC Conjugate)	1:100
Alexa Fluor® 700 anti-human CD56 (NCAM) Antibody	#318315/ BioLegend	10µg/uL per 10^6 cells

Supplementary information

Supplementary Table S2: List of primers used for real time PCR experiment

Primers	Sequences
BST2- F	TCTCCTGCAACAAGAGCTGA
BST2- R	TCTTCTCAGTCGCTCCACCT
DDX58-F	GAAGATCCAGAATGCCAGAATC
DDX58-R	CCACAACCTGTAGGAGCACA
IRF7-F	CATCTTCAAGGCCTGGGCTG
IRF7-R	TTATCCCGCAGCATCACGAA
MAVS-F	CCAGCACCATCCAAATTGCC
MAVS-R	CTCATTTCTGCTGCTCCCGT
β Actin -F	TCCTTCCTGGGCATGGAGT
β Actin -R	AGCACTGTGTTGGCGTACAG
OAS1-F	TCCGTGAAGTTTGAGGTCCAG
OAS1-R	AGGTTTATAGCCGCCAGTCAA
MX1-F	GGAGGCACTGTCAGGAGTTG
MX1-R	TCCTGGTAACTGACCTTGCC
IFI16-F	ACCCGAGAAACAATGACCCC
IFI16-R	AGATGGCTCTCAGGGAAGGT
IFIT1-F	GCCCAGACTTACCTGGACAA
IFIT1-R	TCAAGCACCTTTTCAAAGCA
IFIT3-F	TTGTTGGCCTACATAAAACACC
IFIT3-R	CTGAGAGTCTGCCCAAGTGA
ISG15-F	TGTCGGTGTCAGAGCTGAAG
ISG15-R	GCCCTTGTTATTCCTCACCA
IL6-F	ACTCACCTCTTCAGAACGAATTG
IL6-R	CCATCTTTGGAAGGTTCAGGTTG
IRF-3-F	AAGAAGGGTTGCGTTTAGCA
IRF-3-R	TCCAGAATGTCTTCCTGGGTA
IRF-1-F	GCTGGGACATCAACAAGGAT
IRF-1-R	TGGTCTTTCACCTCCTCGAT

Supplementary information

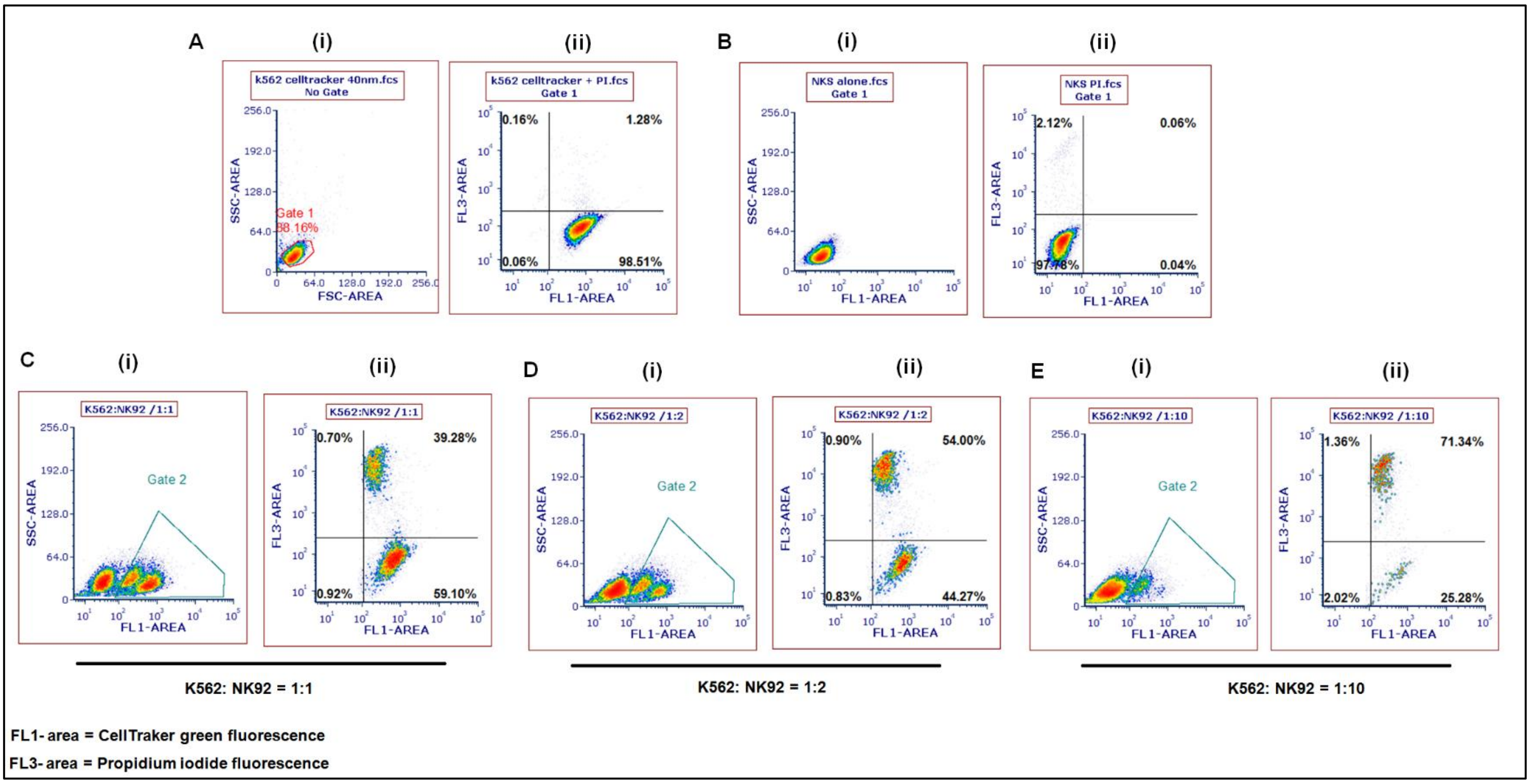

Supplementary Figure S1 - Dot plots representing flow cytometry analysis for the cytotoxic potential of NK-92 cells (effector cells) against K562 cells (target cells).

Briefly, K562 cells, which serve as targets for NK-92 cells, were stained with 40 nM of CellTracker Green probe and incubated for 15 minutes at 37 °C in 0.5% FBS/PBS (pH 7.4). The cells were then washed twice with complete RPMI 1640 and co-cultured with NK-92 cells in RPMI 1640 supplemented with IL-2 (200U) for 4 hours at 37°C. The co-culture reaction volume was maintained at 1 mL. Subsequently, propidium iodide was added at a concentration of 50 µg/mL, and the cells were incubated on ice for 30 minutes before flow cytometry analysis. Individual dye controls were appropriately included for the experiment.

For acquisition in the flow cytometer, we set the FSC/SSC using unstained K562 cells, followed by acquiring K562 with the CellTracker Green probe alone, as indicated in panel A (i & ii). A(i) displays forward scatter (FSC) vs. side scatter (SSC) for the selection (Gate 1) of K562 cells stained with 40 nM of CellTracker Green probe. A(ii) is a dot plot showing fluorescence for K562 cells stained with CellTracker Green probe (FL1- 488/525nm) vs. propidium iodide (FL3-488/630nm). Panel B represents the integrity of NK-92 cells, indicating that the effector cells used in the experiment were healthy. We acquired the NK-92 cells using the same FSC/SSC settings as for K562. The dot plot in panel B (i) shows FL1 (488/525 nm) vs. SSC for unstained NK-92 cells without dye. Panel B (ii) is a dot plot showing fluorescence for NK-92 cells stained with propidium iodide. The upper left quadrant shows the percentage of NK-92 cells positive for propidium iodide (2.12%), indicating that the majority of the effector cells remain healthy. Panels C, D, and E represent the cytotoxicity profiles of the co-culture of K562 cells with NK-92 cells at different ratios. We observe an increase in the death of K562 cells (positive for CellTracker Green and propidium iodide; upper right quadrant) as the ratio of NK-92 cells increases. A 1:1 ratio of K562 to NK-92 cells resulted in 39.28% death, while 1:2 and 1:5 ratios led to 54% and 71.34% death, respectively. Since NK-92 cells effectively target K562 cells, higher ratios of NK-92 cells are expected to increase cell death rates. For analysis, as indicated in C(i), D(i), and E(i), we gated (G2) on cell tracker green positive K562 target cells (FL1; 488/525nm) and then visualized the dead cell percentages by the inclusion of propidium iodide (ex/em 488/630), as shown in C(ii), D(ii), and E(ii).

Supplementary information

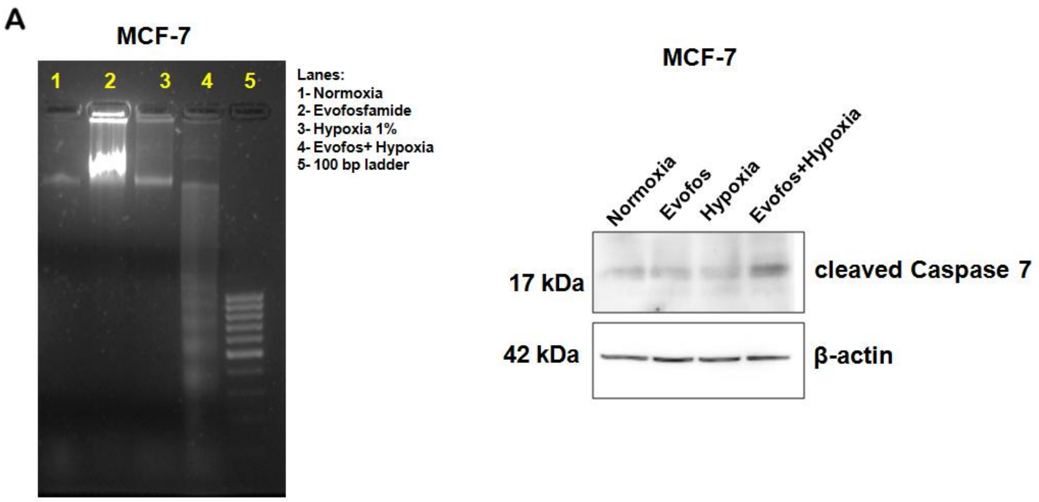

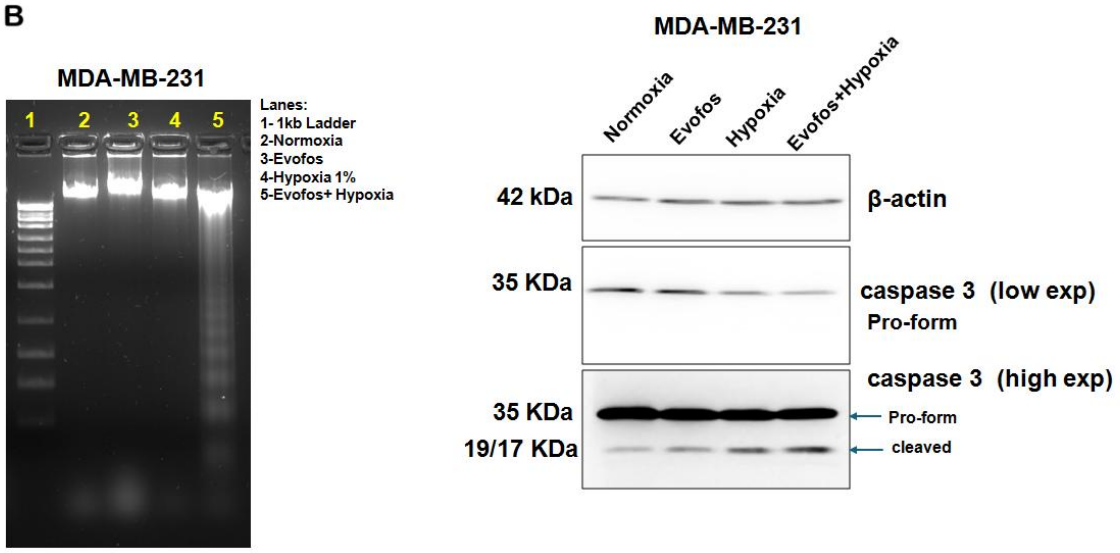

Supplementary Figure S2- MCF 7 cells (A) and MDA-MB-231 cells (B) treated with Evofos under hypoxia exhibit apoptotic cell death, as confirmed by DNA fragmentation assay and immunoblot analysis for caspase 3/7.

Briefly, after treatment with Evofos in normoxia and hypoxia, cells were harvested by trypsinization and lysed in a 1% NP-40 buffer prepared in 50 mM Tris (pH 7.5) and 20 mM EDTA. Next, the lysates were centrifuged at 1600 × g for 5 min at room temperature. The supernatant containing the suspended nuclei was treated with 1% SDS and five µg/µL of RNase A at 56 °C for two hours, followed by proteinase K treatment (2.5µg/µL) overnight at 37°C. Next, DNA was precipitated using 10 M ammonium acetate and absolute ethanol, then dissolved in MilliQ water, and quantified by resolving it in a 1.5% agarose gel with SYBR Safe DNA Gel Stain. The gel images were documented using the iBright CL750 Imaging System (ThermoFisher Scientific, USA). Separately, protein lysates were probed for activation of caspase 7 in MCF-7 and caspase 3 in MDA-MB-231 using western blot.

Supplementary information

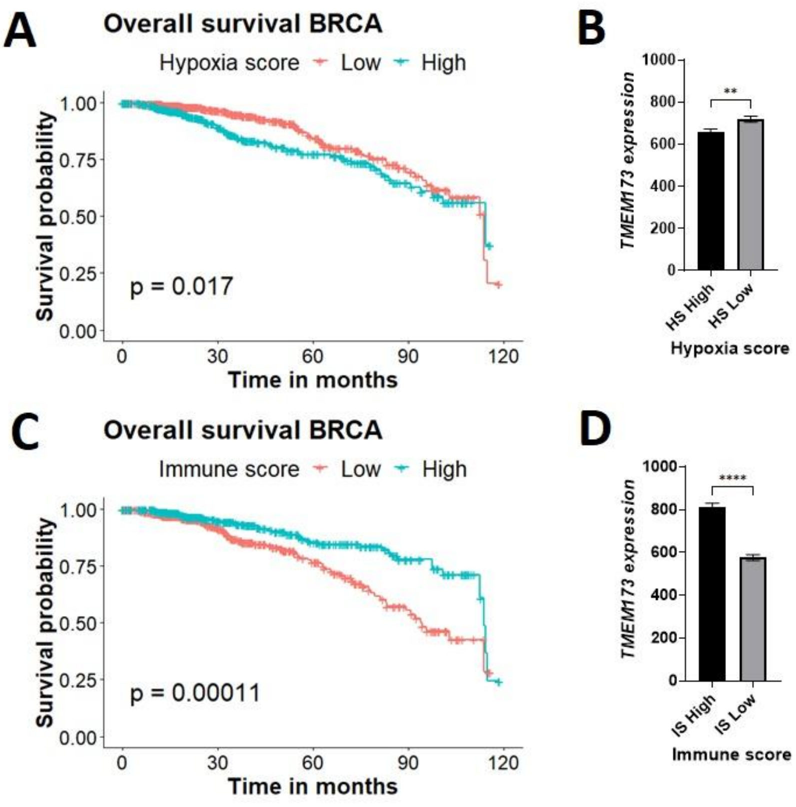

Supplementary Figure S3: Independent sorting of TCGA breast cancer cohorts based on Hypoxia Score (HS) and Immune Score (IS). (A) Kaplan-Meier survival plots compare overall survival between HS High and HS Low. (B) Increased hypoxia score is associated with decreased levels of the TMEM173 transcripts. (C) Kaplan-Meier survival plots compare overall survival between IS High and IS Low. (D) A decreased immune score is associated with lower levels of the TMEM173 transcripts. ** sig-nifies p-value < 0.0021; and *** signifies p-value < 0.0001.

Supplementary information

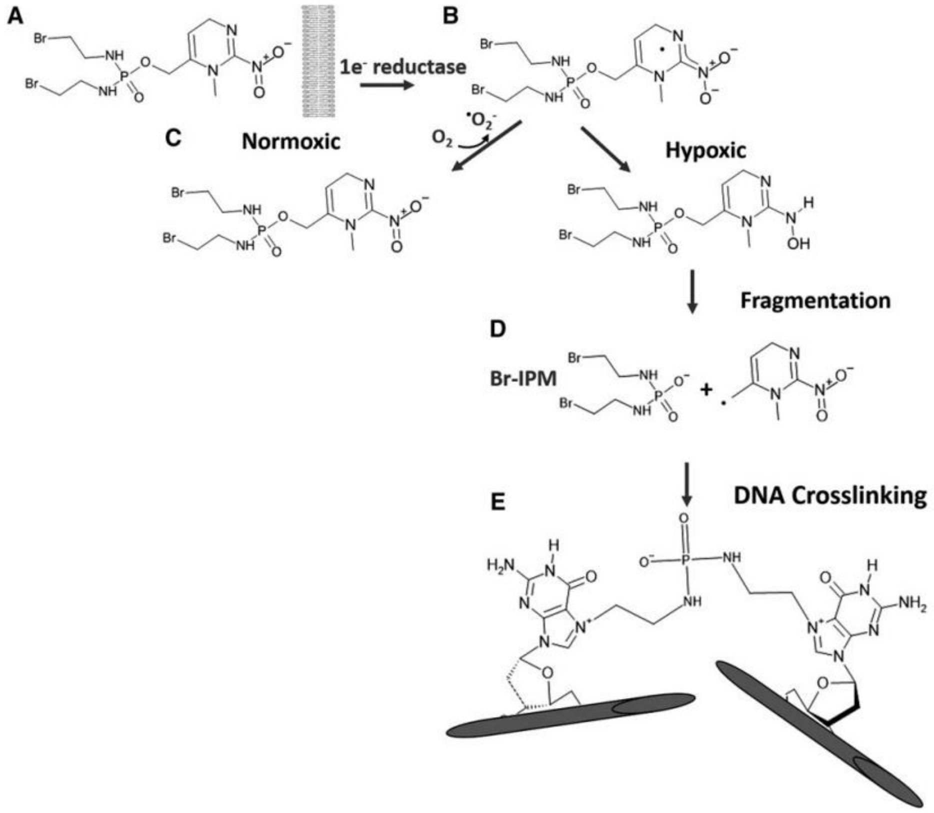

Supplementary Figure S4- Schematics for intracellular conversion of Evofosfamide under normoxia and hypoxia. The image is adapted from Kishimoto et al., 2021. Briefly, Evofosfamide (**A**) is converted to a radical (**B**) in the presence of 1-electron reductases, which can either break down to produce bromo-isophosphoramide mustard (Br-IPM; **D**) under hypoxia that alkylates with DNA, as shown in **E**. Under normoxia, it is believed to convert back to the original prodrug due to the presence of molecular oxygen (**C**). We envision that the conversion of the radical back to the pro-form of the drug (step **B** to **C**) may be altered due to mitochondrial activity.

Reference - Kishimoto et al., Hypoxia-Activated Prodrug Evofosfamide Treatment in Pancreatic Ductal Adenocarcinoma Xenografts Alters the Tumor Redox Status to Potentiate Radiotherapy. Antioxid Redox Signal. 2021 Sep 17;35(11):904–915.
